# Effects of simulated daily precipitation patterns on annual plant populations depend on life stage and climatic region

**DOI:** 10.1186/1472-6785-8-4

**Published:** 2008-03-27

**Authors:** Martin Köchy

**Affiliations:** 1 Research Group Plant Ecology and Nature Conservation, University of Potsdam, Am Neuen Palais 10, 14469 Potsdam, Germany

## Abstract

**Background:**

To improve the understanding of consequences of climate change for annual plant communities, I used a detailed, grid-based model that simulates the effect of daily rainfall variability on individual plants in five climatic regions on a gradient from 100 to 800 mm mean annual precipitation (MAP). The model explicitly considers moisture storage in the soil. I manipulated daily rainfall variability by changing the daily mean rain (DMR, rain volume on rainy days averaged across years for each day of the year) by ± 20%. At the same time I adjusted intervals appropriately between rainy days for keeping the mean annual volume constant. In factorial combination with changing DMR I also changed MAP by ± 20%.

**Results:**

Increasing MAP generally increased water availability, establishment, and peak shoot biomass. Increasing DMR increased the time that water was continuously available to plants in the upper 15 to 30 cm of the soil (longest wet period, *LWP*). The effect of DMR diminished with increasing humidity of the climate. An interaction between water availability and density-dependent germination increased the establishment of seedlings in the arid region, but in the more humid regions the establishment of seedlings decreased with increasing DMR. As plants matured, competition among individuals and their productivity increased, but the size of these effects decreased with the humidity of the regions. Therefore, peak shoot biomass generally increased with increasing DMR but the effect size diminished from the semiarid to the mesic Mediterranean region. Increasing DMR reduced via *LWP *the annual variability of biomass in the semiarid and dry Mediterranean regions.

**Conclusion:**

More rainstorms (greater DMR) increased the recharge of soil water reservoirs in more arid sites with consequences for germination, establishment, productivity, and population persistence. The order of magnitudes of DMR and MAP overlapped partially so that their combined effect is important for projections of climate change effects on annual vegetation.

## Background

Drylands are characterized not only by low water availability but also by great variability of water supply, which play important roles in structuring ecosystems and maintaining biodiversity [1-3]. However, our knowledge about the effects of climate change on vegetation with regard to changes in daily precipitation patterns is still limited [4]. In this paper I use a simulation approach to systematically examine the effect of daily rainfall variability on the growth of annuals in the Middle East along a climatic gradient from arid to mesic Mediterranean.

Precipitation in dry climates has a high variability among and within years [5]. The distribution of annual and daily rain amounts have already changed in the 20th century and are predicted to continue changing [6]. In many Mediterranean regions the number of days in a year with heavy precipitation is increasing, whereas mean annual precipitation is decreasing [7]. For the years 2071–2100, global climate models project a regionally varying shift of both mean annual precipitation and distribution of daily rainfall intensity. For subtropical/warm-temperate regions (25–40° latitude), which include the Middle East, mean annual precipitation is projected to increase by up to 100 mm this century [8]. At the same time mean surface temperatures are projected to increase by c. 1.5°C in tropical and subtropical latitudes (0–35°, [8]). Compared to the change in water supply, the increase in temperature seems less important for plant growth in the arid and semi-arid climates within this range of latitude [4].

The effect of the temporal distribution of water availability on the growth or survival of herbaceous plants has been studied in field and pot experiments in temperate to tropical grasslands [9-13], deserts [14-16], and crops [17,18]. In most experiments the treatments consisted of applying a fixed amount of water in either small amounts with short intervals or in large amounts with long intervals. Generally, the amounts and intervals were uniform during the duration of the experiment. Only the RaMPs project [9,19] varied naturally occurring intervals, although by a fixed percentage. In contrast, climate models and observations suggest that changes will be stronger in the distribution of rainstorms than in the distribution of light rainfalls [7,20]. Experiments that have examined water variability in mesic environments and most studies of crop irrigation methods found that more frequent watering increased plant growth or survival [9,10,14,16,17]. In contrast, studies [13,16,18,21] and simulations [15] related to arid environments show that longer intervals between water pulses can have positive effects when rain pattern and the water holding capacity of the soil interact to produce a longer-lasting soil water reservoir [11,18,22]. The interaction between rainfall variability, soil texture, and water recharge has been described qualitatively by Noy-Meir as the 'inverse texture effect' [1], but the effect of different rain patterns on plant growth in arid and mesic regions has not been studied and quantified yet.

Process-based models represent a good way for studying the presumed causes of the effects of precipitation patterns in greater detail, resolving apparently contradictory effects, and projecting consequences of climate change [4]. Models can be used to vary systematically and in combination characteristics of rain patterns, soil properties, and plant functional types, which would otherwise be hard to achieve logistically or technically in experiments. For example, simulations for North-American deserts have indicated that soil texture, the context of rain events regarding antecedent soil moisture, and the clustering of rain events are important factors controlling plant growth [15].

**Figure 1:**
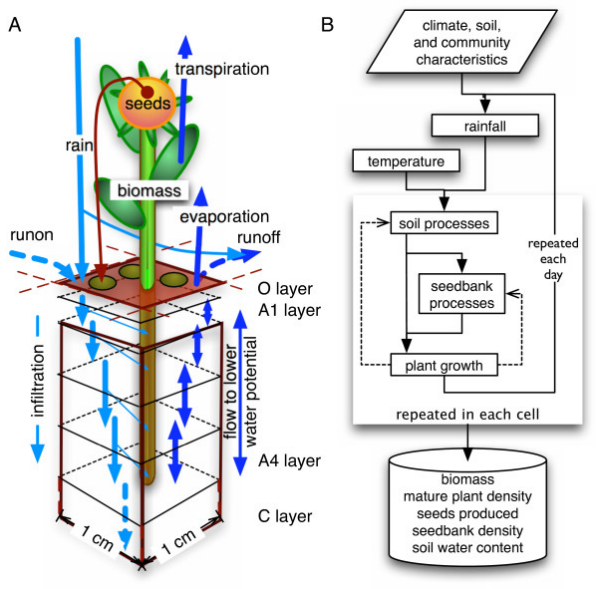
**Graphical representations of the model**. A) Schematic drawing of the simulated processes in one spatial subdivision (cell) of the model. B) Flow chart of the full model. Broken lines indicate feedbacks between modules.

I use a process-based model (Fig. 1) to quantify the effect of different daily rainfall distributions and compare it to the effect of a change in mean annual amount on vegetation. The model simulates explicitly the response of soil moisture and individual plants to rainfall variability. In addition, I consider the effects on different life stages and spatial interactions (competition, dispersal). The model simulates vegetation in the Mediterranean region, situated between water-stressed subtropical and mesic temperate regions. Therefore, the region may be particularly sensitive to climate change effects [23]. Earlier models addressing precipitation effects on Mediterranean vegetation focused on shrubs and trees in the western Mediterranean region [24,25] but did not systematically consider the effect of daily within-year rainfall variability. Annual plants are the dominant or co-dominant life form at lower elevations in the eastern Mediterranean. Furthermore, annuals may respond more directly to water variability than perennials given the annuals' shallower and less plastic root system [26,27]. My model is the first to systematically examine the effect of projected changes in daily rain pattern on natural vegetation along a climatic gradient from arid to mesic. Hence, the sensitivity of different climatic regions to daily rain patterns can be compared explicitly. The gradient is also used by complementary field studies [28-30], which provided the opportunity to combine simulations with field experiments for parameterization and validation of the model [31]. The present study provides a comprehensive examination of net precipitation effects on representative annual communities by investigating soil processes, plant germination, growth, and seed bank dynamics in five climatic regions.

## Results

I assessed the importance of intra-annual rainfall distribution on soil moisture, seedling density, peak shoot mass, and population persistence by varying independently the amplitude of daily mean rain volume (DMR) and the mean annual precipitation (MAP) and comparing their relative effects. DMR and MAP were varied from -20% to +20% relative to current conditions. Daily and thus annual rain amounts were simulated as stochastic time series with specified means. I used relative changes in order to make the effects of DMR and MAP comparable and because my analysis of projections of regional climate models indicated that these factors change relative to the present value. Therefore, results are preferably expressed with regard to the relative change of DMR and MAP. The comparison of effects was conducted for one site in each of five regions (AR, SA, DM, TM, MM) along a climatic gradient in the interior of Israel (Tab. 1). One soil type and one artificial species with representative characteristics to match overall community structure per site were used in the simulations (Additional file 1: ParametersSoil.pdf, Additional file 2: ParametersSeedBank.pdf, Additional file 3: ParametersPlants.pdf). Artificial species differed only in few characteristics (soil moisture required for germination, permanent wilting point, minimum mass for reproduction) to facilitate comparisons. Each site was represented by a grid of 25 × 25 1-cm^2 ^cells (Fig. 1A).

**Table 1:** Standard climate parameters used in the model.

**Climatic region**	**MAP (mm)**	***T*_**a **_(°C)**	***r*(K)**
arid, AR	100	20.5	± 6.5
semi-arid, SA	300	20.0	± 7.0
dry Mediterranean, DM	450	19.5	± 7.0
typical Mediterranean, TM	600	19.0	± 7.0
mesic Mediterranean, MM	800	20.5	± 7.5

MAP: mean annual precipitation, *T*_a_: mean annual temperature, *r*: half-range between coldest and warmest month. Temperature parameters are rounded values of long-term records from nearby meteorological stations.

### Precipitation and soil moisture

Increasing the amplitude of a bell-shaped, seasonal distribution of DMR by 20%, while keeping the mean annual precipitation constant, decreased the average number of days with >5 mm of rain and increased the number of days with >15 mm and > 25 mm. Correspondingly, the median interval between days with >5 mm rain decreased, but it increased between days with >15 and >25 mm rain. Thus, the manipulation of rain pattern shifted the distribution of daily rain volumes to more frequent heavy rainfalls at the cost of light rains as observed in the Mediterranean [7].

The annual mean longest wet period (*LWP*) was assessed as an integrative measure of water availability. *LWP *was defined as the maximum number of consecutive days where the water potential (Ψ) of at least one of the four mineral ("A") soil layers is higher than the permanent wilting point (-3 MPa) representative of the herbaceous plants along the aridity gradient.

**Figure 2:**
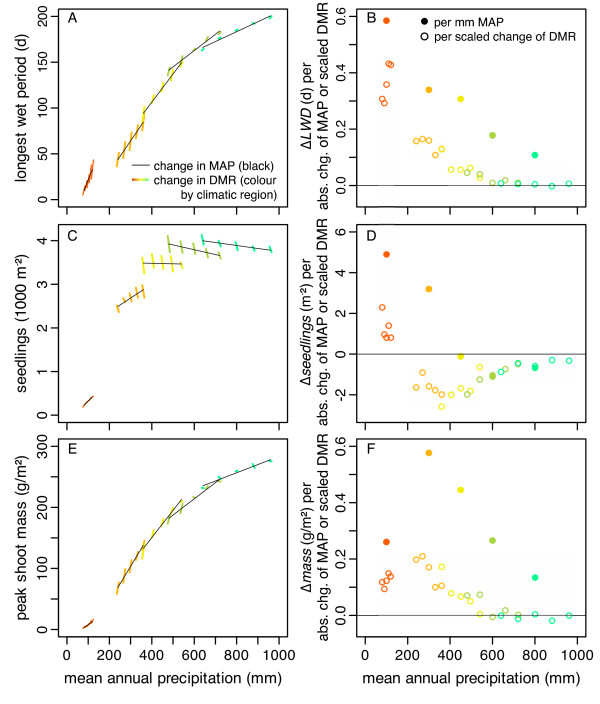
**Change of longest wet period, seedling density, and peak shoot mass with rain manipulations**. Change of (A) longest wet period (*LWP*, maximum number of consecutive days with soil water potential Ψ > -3 MPa), (C) seedling density, and (E) peak shoot mass with mean annual precipitation (MAP, thin/black lines) and with daily mean rain (DMR, thick/coloured lines, 40% change of DMR scaled to 10 mm MAP). (B, D, F) Absolute changes of variables expressed with regard to absolute changes of MAP (range of black lines of Fig. 2A, C, E) [dots, ●] and, for comparison, absolute change of variables per relative change of DMR scaled with MAP [circles, ○]. In other words, the slope of each line in Fig. 2A, C, E was multiplied with the line's mean MAP value. Colours correspond to different climatic regions (red: arid, orange: semiarid, yellow: dry Mediterranean, green: typical Mediterranean, bluegreen: mesic Mediterranean).

*LWP *varied with DMR depending on interactions with regions and regions × MAP (Tab. 2). In addition, *LWP *varied with MAP depending on interactions with regions. The average *LWP *of each region increased in a slightly sigmoid way along the humidity gradient (Fig. 2A). Within sites, *LWP *increased with MAP. The 'absolute' rate of increase (Δ*LWD*/ΔMAP) diminished from the arid to the mesic Mediterranean region (Fig. 2B), whereas the 'relative' rate of increase (Δ*LWD*/10% MAP), indicating the effect of expected relative changes, peaked in the dry Mediterranean region (Additional file 4: RelativeChanges.pdf). *LWP *increased with DMR when MAP was <600 mm. Beyond this point, *LWP *did not change significantly with DMR. The highest relative rate of increase occurred around 200 mm MAP. Thus, the relative rate of increase rose with MAP in the arid region, decreased in the semiarid and dry Mediterranean regions, and did not change with MAP in the two most mesic sites.

**Table 2:** ANCOVAs of *LWP*, seedling density, and peek shoot mass.

**Effect**		***LWP***	**Seedling density**	**Peak shoot mass**
	
	***df***	*F*	*F*	*F*
regions	4	19745	25821	19451
MAP	1	4184	2^n.s.^	3607
DMR	1	269	211	81
regions × MAP	4	74	111	192
regions × DMR	4	23	26	14
MAP × DMR	1	<1^n.s.^	3^n.s.^	10**
regions × MAP × DMR	4	4**	4**	3*
error	605			

*LWP*: longest wet period. n.s.: not significant, *:*P *< 0.05, **:*P *< 0.01, no symbol:*P *< 0.0001.

The results presented here are based on *LWP *calculated for a permanent wilting point of -3 MPa. Wilting points down to -32 MPa, much lower than the conventional -1.5 MPa for cultivated plants [32], are characteristic for dryland annual species [33]. A wilting point of -3 MPa seems representative for a range of annual grass species from dry climates [34]. This includes *Brachypodium distachyon*, a common grass along the precipitation gradient in Israel. The discussion pertains nonetheless to species with low, intermediate, and high permanent wilting points as similar results were obtained for *LWP *calculated for different wilting points of -1.5 and -5 MPa.

### Seedling density

Seedling density was analyzed as an integrative measure of plant responses to DMR via water availability during the early establishment phase. Results reported here, in the preceding and the following section are based on simulations where seed bank density was reset at the beginning of each simulated vegetation year to a value characteristic for each region. The resetting allowed the analysis of rain pattern effects without the influence of inter-annual carry-over effects caused by sequences of wet or dry years. Simulations without seed bank resetting are reported in the section 'Population persistence'.

Seedling density varied with DMR depending on interactions with regions and regions × MAP (Tab. 2). Change of MAP, and its interaction with DMR had no significant effects. The average seedling density of each region increased along the humidity gradient in a hyperbolic fashion (Fig. 2C). Within the arid and semi-arid regions average seedling density increased with MAP, but decreased with increasing MAP in the two most humid regions (Fig. 2D). Increasing DMR at constant nominal MAP increased seedling density in the arid region, whereas increasing DMR generally caused density to decrease in the other regions (Fig. 2D) and did not vary with MAP in the dry Mediterranean region. These contrasting findings suggest that density-dependent germination became more important as water supply increased. The three-way interaction resulted from no effect of MAP on the increase of seedling density with DMR in the arid region, a negative effect of raising MAP on the decrease of density with increasing DMR in the semiarid region, and an on average positive effect of raising MAP on the decrease of density with increasing DMR in the dry to mesic Mediterranean regions.

### Peak shoot mass

The effects of rain manipulations on peak shoot mass were similar to that on *LWP*. Peak shoot mass depended on region, DMR, MAP, and all of their interactions (Tab. 2). The greatest absolute differences among levels were among regions. The average peak shoot mass of each region increased slightly sigmoidally along the humidity gradient. Within each region, mass increased with MAP (Fig. 2E). The 'absolute' rate of increase diminished from the semi-arid to the mesic Mediterranean region (Fig. 2F), whereas the 'relative' rate of increase was greatest in the dry-Mediterranean region (Additional file 4: RelativeChanges.pdf). Further, peak shoot mass (averaged across sites) increased with increasing DMR, but the rate of increase was smaller than that caused by a similar change in MAP. The rate of increase with DMR (averaged across levels of MAP) was greatest in the semiarid region (Fig. 2F). Within the arid region, the effect of increasing DMR was positive and increased with increasing MAP (Fig. 2F). In the other four regions, however, the positive effect of DMR decreased with increasing MAP so that it became insignificant, or sometimes negative, in more mesic regions. In summary, increasing daily mean rain volume and, more strongly, mean annual precipitation both increased peak shoot mass, but their relative effects generally decreased from the semiarid or dry Mediterranean to more arid or more mesic regions.

### Population persistence

**Figure 3:**
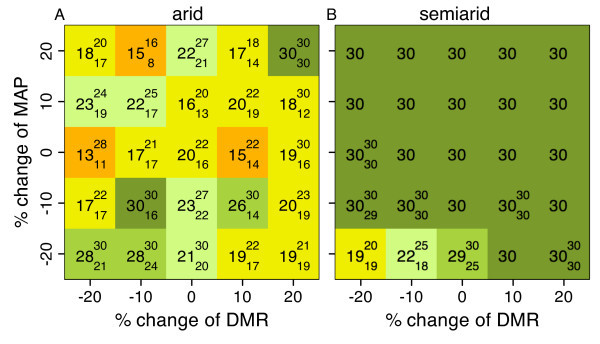
**Persistence of populations in the arid and semiarid regions**. Median persistence (yr) was observed in isolated 25 × 25 cm^2 ^populations. Small numbers represent the lower and upper quartile if persistence of one or more populations was <30 years. Simulations were stopped after yr 30. Colour code: orange: 10–15 yr, yellow: 16–20 yr, pale green: 21–25 yr, light green: 26–29 yr, dark green: 30 yr.

Simulations with a seed bank that carries over to the following year was conducted to assess the consequences of change of rain pattern on population persistence. In the arid region only 25 of 125 populations persisted until the end of the simulations (30 yr, Fig. 3A). In contrast, in the semiarid region 107 populations (Fig. 3B), in the dry Mediterranean region all but one population (combination -20% MAP, -10% DMR), and in the typical and mesic Mediterranean regions all populations persisted for 30 years. In the arid region, populations persisted with full growth cycles only in the combination +20% MAP, +20% DMR. In the other instances, populations survived up to 30 years via a dormant seed bank without germination. Thus, persistence decreased with increasing MAP (*P *= 0.04) and increased with the interaction between DMR and MAP (*P *= 0.001) because persistence was on average higher when both change of DMR and MAP were either both positive or both negative (Fig. 3A). DMR had no significant main effect. In the semiarid region, population persistence increased with DMR when MAP was reduced by 20% (Fig. 3B, *P *< 0.001). Persistence in other combinations and regions was not assessed statistically because the pattern was obvious.

**Figure 4:**
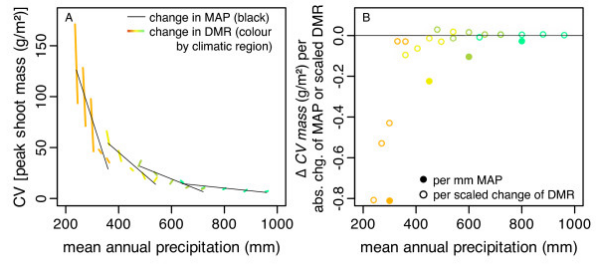
**Temporal variability of peak shoot mass of populations**. A) Change of coefficient of variation (CV) of peak shoot mass with mean annual precipitation (MAP, thin lines) and with amplitude of daily mean rain (DMR, thick lines, 20% change of DMR scaled to 10 mm MAP). B) Absolute changes of CV expressed per absolute change of MAP (filled circles) or MAP-scaled change of DMR (outline circles). The arid region was excluded because most populations did not persist for the full simulation time (30 yr).

I compared the effects of DMR and MAP on the coefficients of variation of annual rain volume (CV_R_), longest wet period (CV_LWP_), and peak shoot mass (CV_mass_) because greater environmental and population variability is theoretically related to shorter persistence [35,36]. I did not include the arid region in this analysis because its CVs were based on less than half the number of years and CV_mass _was strongly influenced by a decline in mean mass over time. CV_R _varied among regions, decreased with change of MAP, and increased strongly with change of DMR (Tab. 3; each *P *< 0.0001). These three factors did not interact. CV_R _could be predicted by the regression equation CV_R _= 19.8 + [3.9 | SA, 1.5 | DM, -1.5 | TM, -3.8 | MM] - 0.084·ΔMAP + 0.128·ΔDMR (adjusted *R*^2 ^= 0.64). CV_LWP _varied strongly among regions (Tab. 3; SA: 53, DM: 31, TM: 17, MM: 12) and decreased overall with increasing MAP. The rate of decrease with MAP within regions became smaller along the humidity gradient. The effect of DMR depended on region. CV_LWP _decreased with increasing DMR across the semiarid region, did not change with DMR across the dry Mediterranean region, increased with DMR across the typical Mediterranean region, and did not change with DMR across the mesic Mediterranean region. CV_LWP _increased with the interaction of MAP and DMR in the semiarid region but in the other regions the interaction effect was not significant. This indicates that water storage in the soil reduced the variability of the rain. The CV of peak shoot mass (CV_mass_) behaved in a similar way as CV_LWP _(Fig. 4; Tab. 3) except that all interactions were significant. CV_mass _decreased along the humidity gradient (Fig. 4A, Tab. 3), with increasing MAP and with increasing DMR. The rate of decrease with MAP became smaller along the humidity gradient. The confidence intervals of the rate of decrease included zero in the mesic Mediterranean region. The average effect of DMR differed among regions because its direction switched from negative to neutral along the humidity gradient (Fig. 4B). The interaction effect on CV_mass _by MAP and DMR was positive on average. In addition, the negative effect of DMR diminished with increasing MAP in the semiarid region only, causing a significant three-way interaction.

**Table 3:** ANCOVAs of the coefficient of variation of rain, *LWP*, and peak shoot mass.

**Effect**		**Annual rain**	***LWP***	**Peak shoot Mass**
	
	***df***	*F*	*F*	*F*
regions (SA-MM)	3	196	1827	603
MAP	1	99	699	621
DMR	1	227	<1^n.s.^	38
regions × MAP	3	<1^n.s.^	52	126
regions × DMR	3	2^n.s.^	19	31
MAP × DMR	1	<1^n.s.^	4*	25
regions × MAP × DMR	3	2^n.s.^	4**	15
error	484			

*LWP*: longest wet period. n.s.: not significant, ***:*P *< 0.001, no symbol:*P *< 0.0001.

The CVs of all three variables were positively correlated among each other (Tab. 4). CV_mass _was generally correlated more strongly with CV_LWP _than with CV_R_. The partial correlation between CV_mass _and CV_R_, accounting for the correlation with CV_LWP_, was negative in the semiarid, dry, and typical Mediterranean regions and neutral in the mesic Mediterranean region. The negative partial correlation indicated that soil water storage (via *LWP*) also reduced the inter-annual effect of rain variability on the variability of shoot mass.

**Table 4:** Correlation between coefficients of variation of annual rain, *LWP*, and peak shoot mass.

	Across regions	SA	DM	TM	MM
CV_LWP _× CV_rain_	0.72	0.38	0.55	0.60	0.73
CV_mass _× CV_rain_	0.55	0.14^n.s.^	0.35	0.46	0.62
CV_mass _× CV_LWP_	0.89	0.82	0.92	0.87	0.85
CV_mass.LWP _× CV_rain_	-0.29	-0.30	-0.47	-0.16	0.01^n.s.^

Values are Pearson correlation or partial correlation coefficients. CV_mass.LWP _× CV_rain_: partial correlation with respect to CV_LWP_. n.s.: not significant (*P *> 0.05).

## Discussion

I simulated the change of stochastic daily rain patterns by increasing the seasonal amplitude of daily mean rain volume (DMR) matched by a decrease in the seasonal occurrence of rainy days so that the mean annual volume remained unchanged. As a result of the increase of DMR, heavy rainfalls contributed more and light rainfalls less to annual precipitation than under current conditions.

### Soil moisture

The shift of rain pattern had clear effects on the average longest wet period (*LWP*; Fig. 2A, B). The effect of changing mean annual precipitation (MAP) was stronger than that of changing DMR and keeping MAP constant but the orders of magnitude were similar. The increase of *LWP *based on *absolute *change of MAP became flatter along the humidity gradient (Fig. 2B) because additional rain was less important for maintaining the soil moisture of an already moist soil in the more humid regions. In addition, the soil became saturated and additional water drained to lower soil layers or ran off at the surface. The increase of *LWP *with *relative *change of MAP was unimodal and greatest in the dry Mediterranean region (Additional file 4: RelativeChanges.pdf) because absolute changes of MAP were smaller in the more arid regions and the effect of increasing MAP diminished with the humidity of the region.

Changing DMR produced a complex response of *LWP*. In almost all instances, a greater amplitude of DMR, *i.e*., more days with rainstorms, prolonged the wet period but the effect size decreased with the humidity of the region. The effect of DMR occurred to a small degree because a higher likelihood of rainstorms advanced and delayed the average dates of the first and last rainstorms, correspondingly, prolonging the wet period. More importantly, rainstorms filled up the soil immediately and to a greater depth than did light rains, especially in the more arid regions with sandier soils. The depth of recharge considered in the model is within the soil's A layer (extending to 16–31 cm, depending on region), where annuals have most of their roots [27] and from where grassland plants take up most of their water [37]. The water in deeper parts of the A layer evaporated more slowly than it would do near the surface, because the soil dried out from the surface downwards and dry soil was less permeable than wet soil. This response of *LWP *to a change in DMR was due to the texture-dependent, non-linear relation between soil water potential and soil water content as shown by the high ranks of retention-curve parameters in the sensitivity analysis (see *Sensitivity analyses*, below). It corroborates other simulations and experimental results [15,22] that sandier soils have deeper soil water recharge from rainstorms than clayey soils, whose water availability is increased by more regular rainfalls (inverse-texture effect [1]). The latter effect did not appear in my simulations because the clayey soils were restricted to the more humid climates, where the mean interval between rainfalls > 2 mm (3 d) was sufficient to keep the soil's A layer above the *LWP *threshold of -3 MPa. The relative effect of soil texture on peak shoot mass is comparable to that of ± 20% change of DMR in simulations [38]. Therefore, the parameters of the soil module should be adapted when simulations are carried out for other target sites. The relation between soil texture and water holding capacity also affects nutrient availability [39], which was not considered in the simulations, and plant growth [22,25,40], which will be discussed farther down.

In summary, the effects of changing MAP and DMR on soil water supply to plants were additive, depended on soil texture, and increased with the aridity of the region. Changes in DMR became important only when they were strong and the change in MAP was small.

### Seedling density

Seedling density increased *among *regions along the humidity gradient (Fig. 2C, D) because the number of seeds was a fixed input parameter that increased from the arid to the mesic Mediterranean region and germination rates did not differ enough among sites to change this pattern. The increase of seedling density with MAP *within *regions was positive in the arid and semiarid regions but negative in the Mediterranean regions (Fig. 2D). This was the outcome of combining the concept of hydrothermal time for germination [41] with density-dependent germination fractions [42] in the model. Evidently, additional rain enhanced the number of days when conditions for germination were met and increased establishment in the arid and semiarid regions. In contrast, more rain in the Mediterranean regions did not greatly improve the conditions for germination. More rain, however, raised the germination fraction (*Detailed model description – Seed bank module*, eq. 2) so that competition among germinating seeds intensified and relative seedling density decreased with increasing MAP in the Mediterranean regions. Test simulations without density regulation showed that heavy rainfalls would cause mass germination in the mesic regions and later strong competition among adult plants. Therefore, density-dependent germination is an evolutionary stable strategy [43]. Empirical evidence for this strategy in natural communities is accumulating [42-47].

Independent of changes to MAP, increasing DMR increased seedling density in the arid region, but decreased density in the other regions. In all regions, germination fractions increased with more regular rainfalls according to the applied hydrothermal time concept, but seedlings had access only to water in the upper 5 cm of the soil, which dried out quickly. Therefore, successful establishment of seedlings in the arid region was mostly controlled by access of seedling roots to water provided by rainstorms (high DMR). The increase of seedling density with less frequent watering was also observed in dry temperate grassland, but the effect was attributed to the breaking of seed dormancy [48]. In the less arid regions more regular rainfalls increased the germination fraction and rainstorms were less important for providing water for seedlings. In summary, seedling density was determined mostly by seed availability, density-dependent germination, number of rainstorms under arid conditions, and regularity of rain under more mesic conditions.

### Peak shoot mass

Generally, the effect of rain manipulations on peak shoot mass (Fig. 2E) followed a similar pattern as that on *LWP *(Fig. 2A), showing the strong control that water as a limiting resource exerted on growth. The change of peak shoot mass with relative change of MAP was greatest in the dry Mediterranean region. There, more seeds could germinate and more rain improved soil moisture conditions during establishment than in the more arid regions. On the other hand, individuals grew more sparsely so that competitive effects were weaker than in the typical and mesic Mediterranean regions. In general, the importance of rainstorms (high DMR) for growth *decreased *via *LWP*, whereas the importance of competition and the density of individuals *increased *along the humidity gradient. This change of ranking also showed in the sensitivity analysis (see *Sensitivity analyses*, below).

The decrease of seedling density with MAP in the non-arid regions did not cause a similar decrease in peak shoot mass. Instead, competition for water among established plants reduced their density. Since the effect of competition decreased with increasing *LWP*, the final density of mature individuals increased in the semiarid to typical Mediterranean regions but still decreased with *LWP *in the mesic Mediterranean region. In this region the decrease of density, however, was compensated by higher production per individual so that peak community shoot mass still increased with MAP.

To summarize, the sigmoid increase of community mass with MAP resulted from a similar increase of *LWP *with relative change of MAP along the humidity gradient, a larger effect of additional rain on establishment in arid than in non-arid regions, and increasing importance of competition for water. The increase of community mass with DMR was the sum of a positive effect of rainstorms on establishment in more arid regions, a negative effect on germination in non-arid regions, and a positive effect on *LWP *resulting in more intense competition but also higher production with increasing MAP.

### Comparison with experiments

The result that the effect of daily rain distribution on the growth of annuals depends on the climatic humidity of the region was also found in experiments where the temporal distribution of water applications was varied independent of the total amount. In more mesic regions, plant growth either remains unaffected or increases with more evenly distributed water applications (lower DMR). For example, in a temperate grassland, water applications every second day vs. monthly more than doubled plant biomass [10]. Similarly, in tall-grass prairie, doubling the intervals between rainfalls (higher DMR) reduced the standing crop by about 10% [9]. Tropical savanna grasses, however, did not respond to watering variance, neither in the field nor in pot experiments (unclipped treatment) [13]. In contrast, plant growth in arid regions tends to be enhanced by stronger pulses with longer intervals (higher DMR). Irrigation every two weeks instead of daily increased the yield of onions by about 40% in sub-Sahelian Burkina Faso [18]. Greater water volumes applied with longer intervals increased the density of annuals and growth of perennials in an arid pasture [21]. The significance of climate was directly shown experimentally [49]. Increasing DMR by increasing intervals between water applications reduced community productivity in a mesic tall-grass prairie but increased it in the drier mixed- and short-grass prairie [49]. The climate effect was also shown in a pot experiment using species of Mediterranean and desert origin [16]. The two desert species grown with low total seasonal water supply had highest survival rates when pulse intervals after establishment were long (11 or 23 d). In contrast, the two Mediterranean species grown with higher total seasonal water supply survived better when time between pulses was 23 d rather than 3 d [16].

Obviously, intervals between rains cannot be extended indefinitely without having a negative effect on plant production. The intervals used in this model (3 d [mesic Mediterranean] - 7 d [semi-arid], 18 d [arid]) are similar to those found in nature [20], where longer intervals between rainstorms are interrupted by light rainfalls. In contrast, 'long interval' treatments in experiments are often regularly spaced and much longer than either natural or projected frequencies for a given location; a factor which may have contributed to the observation of negative correlations between productivity and interval length in mesic climates [9,10,14,16].

### Population persistence

In the second set of simulations, using a dynamic seed bank that included carry-over effects between years, communities in semi-arid to mesic Mediterranean regions persisted for the full simulation time (30 yr) in almost all combinations of change to MAP and DMR. In these regions, the communities produced enough seeds to compensate for low seed production in drought years. The storage effect of the seed bank to buffer environmental variability [50] has also been observed in the field in arid and semiarid ecosystems [51]. Since annual precipitation was not autocorrelated, sequences of drought years were rare and community mass recovered within a few years. Most communities failed to recover in the arid region. Average seed production was not high enough to compensate for drought years. In natural sites in arid regions, populations persist for more than 30 yr because shrubs, crevices, and shade of rocks facilitate the establishment of annuals by indirectly increasing soil moisture [28]. Furthermore, seed dispersal by animals, wind, and surface runoff from these protected sites would also increase community persistence [52]. This continuous replenishment of the seed bank from external sources corresponds to the constant seed bank scenario and underlines the importance of external seed input.

Although populations in the Mediterranean regions persisted for at least 30 years, rainfall variability affected community dynamics across years. The annual variability of peak shoot mass decreased along with the variability of annual precipitation and *LWP *as MAP increased (Fig. 4). Generally, annual rainfall is less variable in more humid climates [5] and variability of grassland production increases with the variability of precipitation [53-55]. Increasing DMR increased the variability of annual rain volume but decreased the variability of *LWP *and peak shoot mass in drier regions and had neutral or positive effects in the more mesic regions. The negative correlation of DMR with the variability of community mass via *LWP *on the decadal time scale was in line with the beneficial effect of DMR on community mass at the annual scale, despite the increase of annual rainfall variability with DMR. This supports Reynolds et al. [15] that in terms of the pulse-reserve hypothesis [1] "pulse" ([*sensu *15] must refer directly to the resource as it is available to the plant and not to the resource as it is supplied. In this case, the pulse must be sought in the soil moisture reservoir and not in the rain.

How does precipitation change projected by global climate models translate into community change along the gradient? The RegCM3 climate model generally predicts a 10 to 20% increase of DMR for the Middle East, but change of MAP varies between -20 and +10% [56-58]. Although the community response to an increase of DMR would have an opposite trend to the response to a decrease in MAP, the latter is much stronger. Consequently, the net effect is negative unless the decrease in MAP is small and the increase in DMR is strong. At sites with soils that cause a negative response of community mass to the increase of DMR, however, the effect of decreasing MAP would be amplified.

### Model extensions and suggestions for further research

The present model concentrates on simulating annuals as one functional group. Apparently, the general results also hold for perennial herbs [49]. Simulating this growth form would require an adaptation of parameters but only small changes to model structure. Vegetative reproduction would have to be introduced and the possibility that one individual may occupy more space than one grid cell. Although herbs take up water mostly from upper soil layers [59], potential uptake from deeper layers could be explored by increasing the parameter for layer thickness or by adding additional layers, requiring just small changes to the soil and plant modules. The model already includes structures for simulating competition for light and nitrogen and interactions with shrubs, although they have not yet been validated. This would allow studying competitive relationships with woody plants in relation to soil moisture distribution [12]. The model can be parameterized for different annual species. Simulations for characteristic species from each climatic region showed that all species responded in a similar way to rain pattern, but some were more sensitive than others [60]. Extending the model to simulate individuals of several species or functional types simultaneously would allow studying effects of rain pattern on, e.g., community composition or invisibility. This would improve our understanding of how different functional types coexist along a gradient of aridity [61]. Specifically, one could test mechanisms for coexistence proposed by a one-dimensional model for plant functional types in deserts [15], especially seasonal differences in water use, differences in preparedness for water flushes, and differences in spatial uptake of water from upper soil layers. The model can also be used in a hierarchical modelling approach [62] to specify the reactions of the herbaceous vegetation for a larger area and coarser scale. For this end I carried out simulations for specific seed bank and rain volume scenarios to obtain transition probabilities of biomass production that were used in models of landscape productivity [63,64].

## Conclusion

Among the populations along the humidity gradient from arid to mesic Mediterranean those in the semi-arid and dry Mediterranean regions were the most sensitive to relative changes in annual precipitation. Changes to daily rain patterns had marked effects on community biomass in more arid regions through the amount of water stored in the soil. The same amount of annual precipitation distributed as sparser, heavier rainfall events recharged the soil water storage in the upper 15–30 cm more effectively than frequent, light rainfall events. Water recharge was increased by a high sand content that increased infiltration rates and reduced hydraulic conductivity in dried out surface layers. In mesic regions changes to daily rain patterns had small or no effects, because the soil remained moist enough between rain events to sustain growth. The effects of daily rain pattern were smaller but of the same order of magnitude as changes to mean annual precipitation. Therefore, these effects should be included in assessments of climate change.

## Methods

### Reference sites

The standard model parameters refer to four experimental sites along a 245-km gradient of humidity in Israel: an arid (AR) site near Sedé Boqér (N 30°52' E 34°46', 470 m a.s.l., 90 mm MAP), a semi-arid (SA) site near Lahav (N 31°23' E 34°54', 590 m a.s.l., 300 mm MAP), a "typical" Mediterranean (TM) site near Matta' (N 31°42' E 35°3', 620 m a.s.l., 540 mm MAP), and a mesic Mediterranean (MM) site near 'En Ya'aqov (N 33°0' E 35°14', 500 m a.s.l., 780 mm MAP). Details of rain fall frequency and distribution at nearby climate stations can be found in Additional file 5: ClimateStations.pdf. The soil types range from sandy loam at the AR site to loam at SA and clay at the MM and TM sites (Sarah Pariente, pers. comm.). Mean annual temperatures vary between 19°C and 21°C. Shrub cover increases along the gradient from <5% (AR) to about 80% (MM). Space between shrubs is covered mostly by annuals, while the percentage of bare ground decreases from about 95% to about 5% along the gradient. The vegetation and experimental setup at the sites are described in greater detail by Holzapfel et al. [28].

The four experimental sites are spread more or less evenly along the humidity gradient with a gap between the SA and TM site. Therefore, I used a hypothetical "dry Mediterranean" site (DM) with interpolated site characteristics in the simulations.

### Model summary

The model (version 2.6.0; Additional file 6: AnnualsModelSourceCode.zip) simulates the effect of rainfall variability on soil moisture and annual vegetation. The model is spatially explicit [65] because it includes the competitive interactions among individual neighbouring annual plants and uses principles of cellular automata models. The model's spatial extent is a soil surface of 25 cm × 25 cm per simulated site, divided into 1-cm^2 ^cells representing soil columns (Fig. 1A). Each soil column contains a seed bank and space for one plant to reach maturity. The model consists of four modules describing the dynamics of climate, soil, seed bank, and plants (Fig. 1B).

I used five parameter sets to represent the different climates, soils, and plant communities in the five modelled climatic regions (Tab. 1; Additional file 1: ParametersSoil.pdf, Additional file 2: ParametersSeedBank.pdf, Additional file 3: ParametersPlants.pdf). The parameters were determined from experiments at each field site, greenhouse experiments, and published data or by choosing values that reproduced field data (for details see *Detailed model description*, below). All modelled processes are defined by physical or logical rules for time steps of one day, except for seed dispersal and seed bank mortality, which are evaluated in annual time steps.

The climate module (for details see *Detailed model description – Climate module*, below) determines daily temperature and precipitation, which are equally distributed in the grid. Temperature is calculated deterministically by a cosine function. Daily values do not vary among years. In contrast, rainfall is generated stochastically by the ReGen algorithm [20] (see below: *Precipitation scenarios*).

The soil module (for details see *Detailed model description – Soil module*, below) simulates water infiltration along a slope. A part of the surface water infiltrates into a soil column that consists of one organic (O) layer and four mineral (A) layers. In the simulations excess rain (runoff) was removed from the grid because I was interested in the direct effect of rainfall volume on each cell and similar moisture conditions across the grid. Water is lost to the atmosphere by evapotranspiration. Soil characteristics like hydraulic conductivity, field capacity, residual (minimum) water content, and the parameters of the soil moisture retention curve [66] are kept constant during simulations and are the same for each layer. Infiltration coefficients decrease with soil moisture and increase with vegetation cover.

The seed bank module (for details see *Detailed model description – Seed bank module*, below) simulates granivory, seed bank mortality, and germination. Before dispersed seeds enter the seed bank, a portion of the seeds in the seed bank dies because of decay, burial, or other causes. A portion of dispersed seeds is eaten before they enter the seed bank if their density is higher than the giving-up density of granivores. I implemented seasonal seed dormancy as an inverse U-shaped function. Germination of germinable seeds is determined by a hydrothermal time approach [41,67] that incorporates the average soil moisture and temperature before germination. The actual germination fraction is further controlled by the local density of seeds [42,43].

The plant module (for details see *Detailed model description – Plant module*, below) simulates the growth of individual annual plants. Plant growth is sigmoid, defined by maximum mass and maximum growth rate. The actual growth rate depends on temperature, water availability, the species' permanent wilting point (PWP), and net size-asymmetric competition [68] with neighbours within a radius of up to 2.5 cm. A fixed portion of produced biomass is allocated to reproduction and converted to seeds when the plant dies. Plant mortality is caused by lack of water. Seeds are dispersed with a negative exponential distribution, *i.e*., the likelihood to disperse farther from the mother plant decreases with distance.

The simulation of soil moisture dynamics and vegetation production by the model was validated by comparison with independent data that were not used to parameterize the model (for details see Additional file 7: Validation.pdf). The sensitivity of the model to changes of the model parameters was tested separately for the abiotic and biotic modules (see Additional file 8: SensitivityAnalyses.pdf).

My model does not take into account increasing annual mean temperatures, CO_2 _concentrations or nitrogen deposition from the atmosphere. A multi-factorial field experiment including these factors and precipitation in an annual grassland showed that interactions among these factors become rare over time and nitrogen deposition produced the strongest effect [69]. Nonetheless, in the long-term, aboveground production of herbaceous arid communities is most strongly correlated with annual precipitation [70], which is also shown by the tight correlation of community biomass with annual precipitation along the climate gradient (see *Validation*, below).

### Precipitation scenarios

I varied the distribution of volume and frequency of daily rainfall for five sets of nominal mean annual precipitation (100, 300, 450, 600, and 800 mm – corresponding roughly to the mean annual precipitation of the field sites) to investigate the effect of the daily rain pattern on the longest wet period (*LWP*), seedling density, and peak shoot mass of populations of annual plants. The change of daily rain pattern was achieved by increasing the seasonal amplitude of daily mean rain volume (DMR) by -20%, -10%, 0%, 10%, and 20% in the ReGen time series generator [20]. Increasing DMR prolongs intervals between rains, reduces the contribution of days with light rains and increases the contribution of rainstorms to the annual rain volume [20]. In order to compare the magnitude of this treatment, I also varied the mean annual precipitation volume (MAP) by -20%, -10%, 0%, 10%, and 20% in factorial combination with DMR. I used relative instead of absolute changes of precipitation parameters because regional climate models indicate that the changes will be tightly correlated with current values and that relative changes are remarkably constant along the precipitation gradient (M. Köchy, unpublished results). For example, the RegCM3 circulation model projects for the pessimistic A2 climate change scenario an average increase of DMR by 3% and a decrease of MAP by 13% for 2070–2100 for Israel North of the Negev desert, whereas the optimistic B2 scenario results in an average increase of DMR by 27% and an increase of MAP by 12% between 31° and 33° N (calculations based on [56-58]).

### Seed bank scenarios

For the assessment of rain pattern effects on *LWP*, seedling density, and peak biomass, seed bank density in each region was reset to a constant value at the beginning of each vegetation year (AR: 2000, SA: 16000, DM: 17000, TM: 18000, MM: 20000 seeds/m^2 ^corresponding to typical values observed in the field). This constant-seed bank scenario facilitates comparisons among years because carry-over effects of seed bank density due to wet and dry years and mortality are excluded. In a second set of simulations I examined the effect of change of daily rain pattern on population persistence. For population persistence inter-annual seed bank dynamics carry-over effects are important and were included (dynamic-seed bank scenario). To quantify community persistence I recorded the year in which the seed bank had dropped to zero or whether the population survived for the total simulated period.

### Statistical analyses

I simulated 30 years of stochastic rainfall for each combination of levels of DMR and MAP in each climatic region and both seed bank scenarios. This was repeated five times for each combination. I used different random spatial distributions of soil surface heterogeneity in each simulation run. The great detail simulated by the model prevented me from storing daily data and only annual data across the whole lattice was recorded. Therefore, I sometimes phrase interpretations of results cautiously where omniscience of causality in the model could be expected. Among the many vegetation and soil variables simulated by the model, I concentrated on formally analyzing the effect of rain pattern on annual longest wet period (*LWP*), seedling density, and peak shoot mass (maximum annual aboveground biomass) because each is an integrative measure of, respectively, available soil moisture, establishment, and community performance. The effect of daily rain pattern on *LWP*, seedling density, and peak shoot mass for the constant-seed bank scenario was examined with analyses of covariance (ANCOVA) across regions. The full-factorial ANCOVA used change of DMR and change of MAP as covariates and regions as a nominal factor. As the data were very heteroscedastic, I used the means across years for each combination of daily pattern × mean annual precipitation × climatic region × repetition. Slopes and means were compared post-hoc using 95% confidence intervals. In addition, I calculated the change of peak shoot mass per 10% change of DMR and per 10% change of MAP to facilitate the comparison of effect sizes.

In the dynamic-seed bank scenario few populations persisted for the total simulation time (30 yr) in the arid region in contrast to the more humid regions. Therefore, an ANCOVA of persistence comprising all sites as for the constant seed bank scenario was not meaningful and I analyzed the arid region only. This turned the ANCOVA for the arid region into a multiple regression. For the other four regions, where time of persistence was almost always the full 30 years of the simulation runs, I compared the coefficient of variation of annual rain volume, longest wet period, and peak shoot mass using an ANCOVA to test how changes in rain pattern affected environmental and production variability. In addition I tested whether the CVs were correlated.

### Detailed model description

#### Climate module

The climate module generates temperature and precipitation for each day *d*. Mean daily temperature *T*_d _is a function of a seasonal index (*S*): *T*_d _= *T*_a _+ r·*S*, where *T*_a _is the mean and *r *half the range of monthly mean temperatures (Tab. 1). Index *S *is defined as a cosine function with maximum in mid July, minimum in mid January, and starting on August 1 when rainfall probability is lowest along the climatic gradient. Thus, *S *= **cos**([*d *+ *d*_m_]·2*π*/365.25) with *d*_m _= 15.625.

Stochastic time series were determined by the ReGen algorithm [20], a so-called two-part model [71]. It uses a regular bell-shaped seasonal function that determines the daily mean likelihood averaged across years that a given day of the year is a rainy day. A flattened bell-shaped seasonal function determines the daily mean rain volume (DMR), *i.e*., the mean rain volume for a given day of the year averaged for rainy days across years. The DMR for a given rainy day of the year is used as the mean of a random negative exponential distribution from which the rain volume on that day is drawn. The parameters of each of the two seasonal functions were determined from monthly aggregated historic data from climate stations with 100 to 800 mm MAP in the interior of Israel. The time series do not include autocorrelation between annual rain volumes because autocorrelation in the historic data was not significant. ReGen allows the manipulation of DMR by ± 30%. In order to keep MAP at the specified value, the change of DMR is compensated by decreasing the occurrence of rainy days

#### Soil module

**Figure 5:**
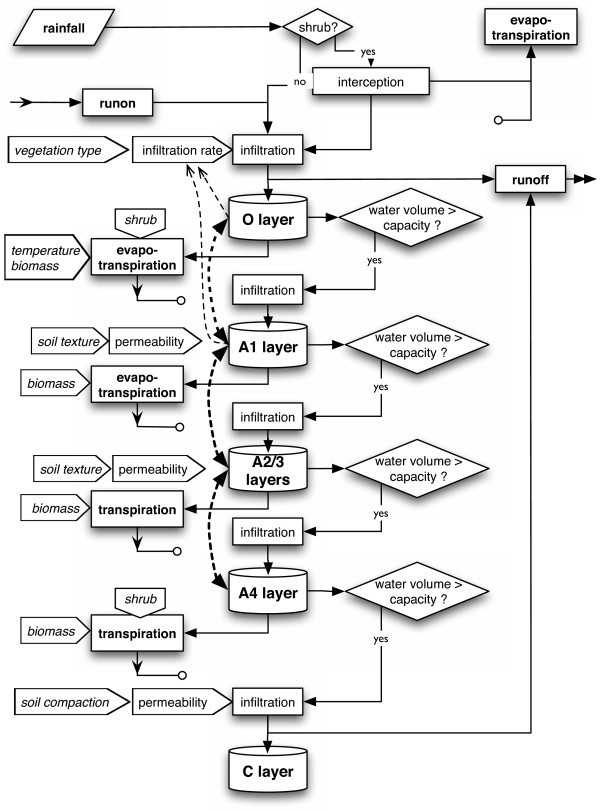
**Flow chart of the soil module**. Broken thick lines indicate water flow due to differences in water potential. Broken thin lines indicate feedbacks from soil moisture on infiltration rate.

The soil module determines the water availability for plant growth by simulating water infiltration, surface runoff, and evapotranspiration of individual soil columns with a surface of 1 cm^2 ^(one cell; Figs. 1, 5) with different standard parameters for each region (Additional file 1: ParametersSoil.pdf). A soil column consists of one organic ([O layer] 0 – 1 cm) and 4 mineral soil layers (A1: 1 – 5 cm, A2: 5 – 10 cm, A3: 10 – 15 cm, A4: 15 cm down to the weathered mineral layer [C layer] at 16 to 30 cm depending on region).

Rainfall distribution at this spatial scale is assumed to be equal, thus all soil columns receive the same amount of rain. I assume that surface unevenness like small depressions or individual stones randomly change water reaching the soil by a fraction of ± *H*%. This fraction *H *is constant for each cell across years in each simulation run. If, however, the rainfall is >10 mm/day, I assume that this unevenness has no effect. Water that has reached the soil surface partly infiltrates the soil, the rest (runoff) is removed from the cell. Runoff in the model was removed in order to exclude effects on soil water due to relative location on the simulated grid. The amount of infiltrating water is calculated as volume_in _= [*IC*_dry _- *v*·Δ*IC*·**min**(*θ*_A1_, *θ*_A2_)]·volume_surface_, where *IC*_dry _is a soil-specific infiltration coefficient (infiltrated volume/daily rain volume), *v *is a fitted parameter accounting for higher soil infiltration in vegetated (*v *= 0.9) than in bare soil (1.0) [72], Δ*IC *is the difference to the infiltration rate in a saturated soil, and *θ *is the volumetric water content of the soil. Thus, the infiltration coefficient decreases as the upper two A layers become wet. I obtained *IC*_dry _by fitting the model output for annual runoff to the mean annual value measured in 0.15 m^2 ^runoff plots at each site in October 2002–April 2003 (Sarah Pariente & H. Lavee, unpublished data). Parameter Δ*IC *was calculated from runoff experiments along an aridity gradient near Jerusalem [73].

Infiltrating water fills up the soil downwards layer by layer. This tipping-bucket approach is sufficient for simulating water distribution in the soil after one day [74]. Each layer cannot contain more water corresponding to a soil water potential Ψ of -0.01 MPa, that is, between field capacity (-0.036 MPa) and saturation (0 MPa). This value was suggested by measurements of the A1 layer at the typical and mesic Mediterranean field sites. The change of soil properties between two layers can reduce the permeability *P *of the soil. In the model this is implemented by letting only a fraction of water (*P*_c_) flow from the A4 into the C layer. This becomes important only in the mesic Mediterranean region where the soil of the field site is compacted (H. Lavee, pers. comm.). There, I assume a permeability *P*_c _of 70%. At the other sites, the C layer is more porous and I assume 100% permeability in these regions. Processes in the C layer are not considered in the model.

If the water remaining in the A4 layer is greater than its capacity, excess water will back up and fill the upper layers to their capacity and, if there is still excess water, contribute to surface runoff. Thus, runoff in the model can be generated in two different ways, infiltration excess (Hortonian runoff) and saturation excess. The model does not provide a mechanism for interflow (water movement in the soil parallel to the surface), because I assume that lateral differences in soil moisture are much smaller than vertical ones.

Analyses of daily soil moisture in the O and A1 layer after heavy rainfalls followed by a dry spell at each of the experimental sites (Sarah Pariente & H. Lavee, unpublished data) and measurements of similar soils [75, Taylor et al. in 76] indicated that evaporation from the A1 layer can be represented by a linear decrease of soil water potential Ψ with time as Ψ_d+1 _= *E*_A1_·Ψ_d_, with daily evaporation factor *E*_A1 _ranging between 2 and 3, depending on site. Evaporation from the O layer was also linear but its evaporation factor *E*_O _(≈3) increased linearly with mean daily temperature *T*_d _(Ψ_d+1 _= *E*_O_·*T*_d_·Ψ_d_). Transpiration by plants is simulated by transpiration factors (Tab. 5) that increase the evaporation factors, differ among soil layers, and vary with aboveground mass to reflect the influence of root distribution. I assume that transpiration factors do not vary among regions because grasses were the dominant annual growth form at each field site.

**Table 5:** Daily change of simulated soil water potential by transpiration depending on aboveground mass.

		Total shoot mass (*m*_t,d_)		
Soil layer	Depth	>10 mg	>100 mg	>200 mg

O	0–1 cm	no effect of transpiration		
A1	1–5 cm	1.05 Ψ	1.1 Ψ	1.2 Ψ
A2	5–10 cm	1.1 Ψ	1.3 Ψ	1.4 Ψ
A3	10–15 cm		1.3 Ψ	1.4 Ψ
A4	15 cm – C			1.2 Ψ
C	>16 ... >30 cm	no effect of transpiration		

Ψ: soil water potential (MPa). Thickness of the A4 layer differs among regions (see additional file 1: ParametersSoil.pdf).

Beyond the quick process of infiltration driven by gravitation potential, I also consider the slower process of water movement driven by differences in water potential in the O and A layers. This water flux is limited by the moisture- and texture-dependent hydraulic conductivity of the soil [77]. In the model, I approximate this non-linear process by first calculating the amount of water (Δ*V*) necessary to change the potential of the thinner of two layers to their geometric mean weighted by layer thickness. This amount is reduced by a conductivity factor *K*_A _that varies with soil texture as $\Delta V=K_{A}\cdot \left(V_{\bar{\Psi }}-V_{\Psi_{\text{thin}}}\right)$. This semi-mechanistic approach prevents a drier thinner layer from becoming saturated or from drying out too much before a moister thicker layer's potential has balanced. The change of water content is calculated layer by layer downwards. As a result of balancing the potential, evaporation sucks water upwards, water reaches previously dry, lower layers, and the potentials of adjacent layers become more similar over time. The mechanism also reproduces the positive correlation between soil conductivity and water content [77], resulting in a lower evaporation from the top layer when the soil is dry than when it is wet. Water does not flow between the A4 and C layer by this mechanism.

To calculate evaporation and water movement, volumetric water content in each soil layer is converted to water potential using a soil moisture retention curve according to van Genuchten [66]:

Ψ=[(θS−θRθ−θR11−1/β−1)1β]⋅α−1

where *θ *is the actual soil moisture (cm^3^/cm^3^), *θ*_S _soil moisture at saturation, *θ*_R _residual moisture, and *α *and *β *are parameters describing the curvature of the function. In the model I use a different set of parameters for each region (Additional file 1: ParametersSoil.pdf). *α *and *β *were fit to measurements of moisture and water potential on similar soils along a gradient of aridity (Sarah Pariente, unpubl. data). For *θ*_S _I used a value slightly higher than the highest soil moisture actually observed at the experimental sites. *θ*_R _was estimated based on values for standard soils [77]. Soil water potential was limited to range between 0 (saturation) and -150 MPa (Ψ_min_), *i.e*., I disregard situations of ponding and extreme desiccation.

#### Seed bank module

**Figure 6:**
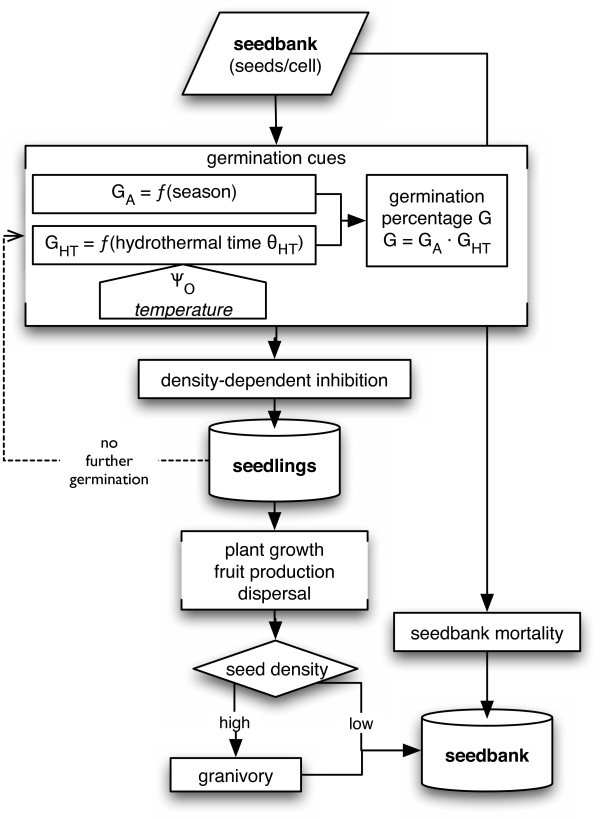
**Flow chart of the seed bank module**. G_A_: 'activity' (inverse of dormancy), G_HT_: 'hydrothermal' germinability (inverse of quiescence), Ψ_O_: soil water potential at 0–1 cm depth.

**Figure 7:**
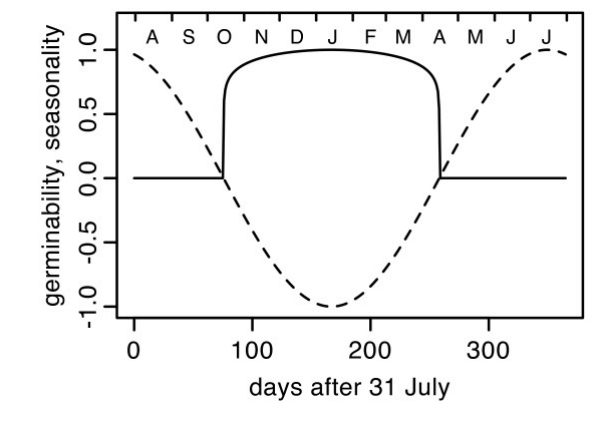
**Time course of germinability and seasonality index**. Continuous line: germinability, dashed line: seasonality index *S*.

The seed bank module simulates the dynamics of the seed bank by germination and seed mortality in annual time steps each summer (Fig. 6) with different standard parameters for each region (Additional file 2: ParametersSeedBank.pdf). Before dispersed seeds enter the modelled seed bank, the seed bank is decreased by 10% to reflect seed mortality, downward movement of seeds in the soil, and other processes that reduce seed viability. Most newly dispersed seeds in the field are eaten by ants or rodents [78,79]. Rodents stop searching for seeds when their density is below a giving up density [80]. I also assume a giving-up density for ants although evidence is inconsistent [81,82]. I assume in the model that 25 – 35% of the seeds survive granivory and that granivores leave a cell when the seed density is <20'000/m^2^. These values were tuned so that simulated populations with unmodified regional parameters persisted for several decades. All seeds in the seed bank are considered viable but dormant at the beginning of the vegetation year (August). In the research area, germination events are observed only from October to March. Germinability of any species is highest in January (K. Tielbörger, J. Kigel, pers. comm.). In the model, these relations are expressed as an inverse-U shaped seasonal function of germinability *G *= (-*S*)^0.1 ^if *S *< 0, else *G *= 0 (Fig. 7), where *S *is the seasonal index of the climate module.

The number of seeds per cell ready to germinate on a specific day (germinable seeds) is determined by their accumulated "hydrothermal time", *θ*_HT _[67], a concept related to temperature sum or degree-days. Hydrothermal time is defined as *θ*_HT _= ∑([Ψ_O _- Ψ_b_]·[*T*_d_-*T*_b_]), where Ψ_O _is the soil water potential in the top layer, Ψ_b _is the minimum water potential required for germination, *T*_d _is mean daily temperature, and *T*_b _is the species-specific minimum temperature required for germination. Hydrothermal time is accumulated daily if the increment is positive. As a strategy to prevent untimely germination, I assume that hydrothermal time is reset to zero if the increment has not been positive for more than seven days. When *θ*_HT _is greater than a species-specific threshold, the seed is ready to germinate. If a cell contains more than one seed, the germination fraction (*g*) is defined as

$$\text{probit}(g)=\left(\frac{\Sigma (\Psi -\Psi_{\text{b}50})}{t}-\frac{\theta_{\text{HT}}}{\Sigma (T_{\text{d}}-T_{\text{b}})}\right)/\sigma (\Psi_{\text{b}}),$$

where Ψ_b50 _is the mean Ψ_b _of the seeds with standard variation *σ*(Ψ_b_). Mean minimum water potential Ψ_b50 _has been found to vary among species [83], populations [84], and sites [41], whereas *T*_b _is assumed mostly constant within species [85]. Germination fraction of annuals in semi-arid regions is strongly density dependent [42,43]. This is incorporated in the model by calculating the maximum seedling density (*D*_max_) within a distance of two cells around a target cell (18 neighbours) on a hexagonal lattice as *D*_max _= *N*^(*a *± Δ*a*)^, where *N *is the mean number of germinable seeds per square metre in the neighbourhood, *a *is the density dependence (≈0.5...1.0 [42]) with random uniform variation Δ*a*. Which of the germinable seeds in a neighbourhood will germinate is decided by chance with the probability adjusted to match *D*_max_. Germinated seeds prevent the germination of other seeds in the same cell so that only one plant can establish per square centimetre at maximum density. This conforms to plant densities observed in the field (pers. obs.) and generates a permanent seed bank.

#### Plant module

**Figure 8:**
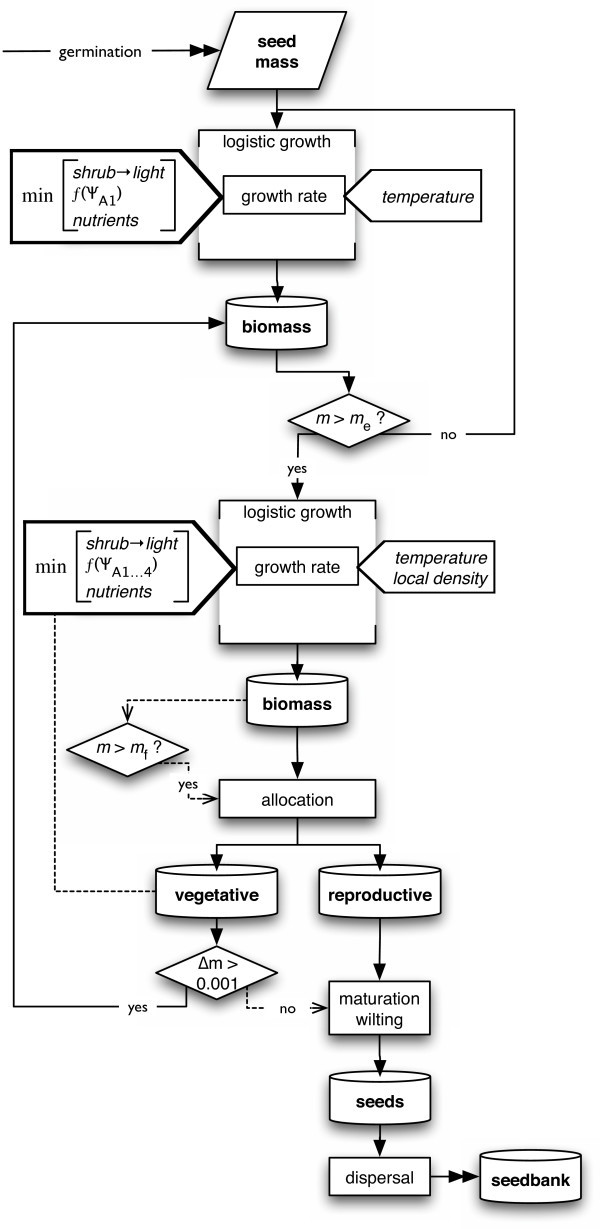
**Flow chart of the plant module**. *d*: day, *m*: mass, *m*_e_: threshold for establishment, *m*_f_: threshold for reproduction, Ψ_A1_: soil water potential in the Al layer.

**Figure 9:**
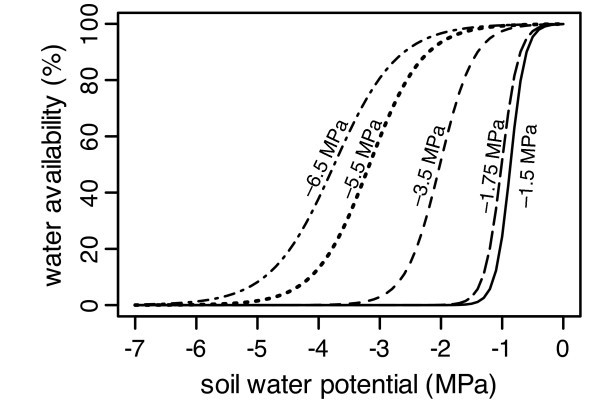
**Relative water availability as a function of soil water potential**. The figure shows curves for different artificial species with representative permanent wilting points of -6.5, -5.5, -3.5, -1.75, and -1.5 MPa.

The plant module simulates the daily growth and seed production of annual plants dependent on water availability and competition (Fig. 8) with different standard parameters for each region (Additional file 3: ParametersPlants.pdf). Growth of germinated seeds starts with seed mass and increases logistically with relative growth rate on day *d*(*RGR*_d_) reduced by a function of temperature (*f*(*T*_d_)), relative resource availability (*RA*_d_), and competitiveness (*C*) as

daily increment Δ*m *= *m*_v,d_·**exp**[*RGR*_d_·*f*(*T*_d_)·*RA*_d_·*C*^2^]

with *RGR*_d _= *RGR*_max _- *RGR*_max_·*m*_t,d_/*m*_max_

with *m*_v,d_: vegetative aboveground mass, *m*_t_: total aboveground mass, *RGR*_max_: maximum relative growth rate and *m*_max_: maximum attainable total mass per individual. *RGR *is halved for every 10 K when the temperature drops below 25°C, reflecting the effect of temperature on biochemical growth processes. Daily relative resource availability *RA*_d _equals water availability (*WA*) in this study because I consider only patches between shrubs with full sun and assume that water is the only limiting resource in this situation. Effective rooting depth of herbs is assumed restricted to the A layer [27,59,76]. Water availability is calculated as a sigmoid function of soil water potential ranging from 0% at the species-specific permanent wilting point (*PWP *= -1.5 MPa for mesic region plant species, -6.5 for arid region species, based on [86]) to 100% at field capacity (-0.036 MPa) as *WA *= 1/(1 + **exp**(*r*/*PWP*·(Ψ - *PWP*) + *s*)), with fitted parameters *r *= 12.5 and *s *= 5.3 (Fig. 9). I assume that the highest water potential of the four A layers controls the growth rate and that access to deeper soil layers increases with plant mass. These categories were estimated and adjusted to fit final community biomass in 2002/2003.

Competition reduces resource uptake und thus the relative growth rate (eq. 3a). I use a simplified field-of-neighbourhood approach [87,88] to simulate net size-asymmetric competition [68]. Each individual is assumed to acquire above- and belowground resources from its 'home' cell, and the 18 surrounding neighbour cells in a hexagonal lattice if the individual's mass is greater than an upper critical mass (*m*_C2_); else, the individual can only access the six adjacent cells. If the mass is smaller than a lower critical mass (*m*_C1_), the individual's uptake is restricted to its home cell. The maximum area of 19 cm^2 ^(19 cells) is 2–3 times greater than the average area per mature individual (7–9 cm^2^; Hadas Parag, unpublished data) in the SA, TM, and MM field site in spring 2003. (The average area per mature individual in the AR site was 255 cm^2^.) Although the radius of this area of potential interaction is smaller than the average lateral spread of roots [27] or shoots of annuals, it will likely include the zone of strongest interaction. For simplicity of programming I assume that each neighbour cell has the same resource availability as the home cell. Resource uptake from the home cell is exclusive to the resident individual but proportional to the individual's competitiveness (*C*) for belowground resources, ranging from 0 to 100%. This means that a plant is most competitive if it takes up resources only from the home cell, where it cannot be suppressed by neighbours. Resource uptake from each unoccupied neighbouring cell is relative to the resident individual's mass compared to the total mass of plants sharing access to the neighbour cell. If competition reduces relative resource uptake below 40% (density-dependent mortality, *M*_D_), the plant dies. Competitive ability is made to have a size-asymmetric effect on growth rate by using its square in eq. 3a.

For a mature plant with *m*_t,d _> *m*_mature_, a fixed portion of the daily produced aboveground biomass (Δ*m*) is allocated to flowers and seed production without contributing to new vegetative growth. *m*_mature _is increasing along the humidity gradient because individuals in arid regions are smaller and often flower and reproduce at smaller size than in more humid regions [89]. Plants are assumed to wilt when their daily production is < 1‰. This may be caused by drought, competition, or attaining maximum mass *m*_max_. The reproductive biomass of wilted plants is converted to number of seeds by dividing by fruit mass. Here, 'fruit mass' is the average mass of reproductive structures per seed in a species.

The number of seeds dispersed from a plant generally decreases with distance. Most annuals in the study region lack a specialized mode of dispersal, their seeds just fall off the plant. In the model, I assume that seeds are dispersed into neighbouring cells with random direction and a mean distance of 5 cm with a negative exponential distribution according to consensus estimates for dominant species by several field experts (C. Holzapfel, M. Sternberg, M. Petrů, pers. comm.).

## Authors' contributions

MK designed and wrote the program, planned and evaluated the simulations and scenarios, and wrote the manuscript.

## Supplementary Material

Additional file 1 **Standard parameters used in the soil module**. Standard parameters used in the soil module for all simulations. Click here for file

Additional file 2 **Standard parameters used in the seed bank module**. Standard parameters used in the seed bank module for all simulations. Click here for file

Additional file 3 **Standard parameters used in the plant module**. Standard parameters used in the plant module for all simulations. Click here for file

Additional file 4 **Absolute changes of variables**. Alternative view of data shown in Fig. 2 based on relative changes of mean annual precipitation. Click here for file

Additional file 5 **Precipitation distribution at climate stations**. Precipitation distribution at climate stations close to the field sites. Click here for file

Additional file 6 **Model source code**. The archive contains directories with the source code in the c++ language distributed over several text files. The archive also includes a sample input file for running the model in batch mode. Click here for file

Additional file 7 **Validation**. The file describes how the soil, seed bank, and plant modules of the model were validated. The file contains text and three figures. Click here for file

Additional file 8 **Sensitivity analyses**. The file describes the method and results of testing the sensitivity of runoff, soil moisture, and peak shoot mass to changes in parameters. The file contains text and two figures. Click here for file
