# Supplementary material for: Effects of simulated daily precipitation patterns on annual plant populations depend on life stage and climatic region
Source: BMC Ecol. 2008 Mar 27;8:4. doi: 10.1186/1472-6785-8-4 (PMC2359731; doi:10.1186/1472-6785-8-4)
Supplement: Additional file 1 — Standard parameters used in the soil module. Standard parameters used in the soil module for all simulations. [file 1472-6785-8-4-S1.pdf]

Standard parameters used in the soil module.

climate	arid	semi-arid	dry Mediterranean	typical Mediterranean	mesic Mediterranean	range in sensitivity analysis
soil texture (1)	sandy loam	loam	clayey loam	clay	clay	
moisture retention (2)						
θ_S (v/v)	0.35	0.4	0.45	0.5	0.55	[0.3, 0.7]
θ_R (v/v)	0.015	0.03	0.04	0.04	0.05	[0.01, 0.1]
α	62	59	84	108	78	[20, 1600]
β	1.38	1.27	1.26	1.25	1.22	[1.1, 1.8]
depth A4 layer (cm) (3)	1	5	10	15	10	[1, 15]
infiltration rate (4)						
I_{dry} (%)	92	93	94	100	99	[70, 100]
ΔI	–36	–29	–20	–1	–9	–[0, 40]
hydraulic conductivity						
K_A (%)	30	50	60	70	60	[30, 100]
permeability P (%) (4)	100	100	100	100	70	[50, 100]
evaporation rate						
E_O	$3.0 \cdot T$	$3.0 \cdot T$	$3.0 \cdot T$	$3.0 \cdot T$	$3.0 \cdot T$	$[1.5, 4] \cdot T$
E_{A1}	3.0	3.0	2.5	2.5	2.0	[1.5, 4]
surface heterogeneity H (%)	90	50	20	10	10	[0, 100]

T: daily temperature (°C)

(1) Sara Pariente, pers. comm.
(2) van-Genuchten parameters derived from water content measurements at similar sites (Sara Pariente, pers. comm.)
(3) H. Lavee, pers. comm.
(4) derived from runoff measurements at the field sites (H. Lavee, unpublished results, 2004)
