# Supplementary material for: Effects of simulated daily precipitation patterns on annual plant populations depend on life stage and climatic region
Source: BMC Ecol. 2008 Mar 27;8:4. doi: 10.1186/1472-6785-8-4 (PMC2359731; doi:10.1186/1472-6785-8-4)
Supplement: Additional file 2 — Standard parameters used in the seed bank module. Standard parameters used in the seed bank module for all simulations. [file 1472-6785-8-4-S2.pdf]

Standard parameters used for seed germination.

climate	arid	semi-arid	dry Mediterranean	typical Mediterranean	mesic Mediterranean	range in sensitivity analysis
representative species (for illustration only)	*Stipa capensis*	*Crithopsis delileana*	*Trisetaria macrochaeta*	*Avena sterilis*	*Brachypodium dystachium*	
seed bank density (seeds/m^2)	500 (1)	11'000 (1)	15'000	17'000 (1)	30'000 (1)	[500, 50'000] (1)
range (%)	±50 (1)	±11 (1)	±11	±11 (1)	±11 (1)	
seed bank persistence (%)	90	90	90	90	90	
survival of dispersed seeds (%)	30±5	30±5	30±5	30±5	30±5	
granivory threshold (seeds/m^2)	20'000	20'000	20'000	20'000	20'000	
hydrothermal time						
Ψ_{b50} (MPa)	–1.03 (3)	–0.87 (3)	–1.00	–0.86 (3)	–0.67 (3)	[–1.5, 0.5] (2)
σ (MPa)	0.5 (2)	0.5 (2)	0.5 (2)	0.5 (2)	0.5 (2)	[0.2, 0.9] (2)
T_b (°C)	0 (3)	0 (3)	0 (3)	0 (3)	0 (3)	
θ_{HT} (MPa·K·d)	25 (3)	31 (3)	30 (3)	28 (2)	23 (3)	[12, 130] (2,3)
dry days	7	7	7	7	7	[5, 10]
density regulation						
a	0.77 (4)	0.77 (4)	0.77 (4)	0.77 (4)	0.77 (4)	[0.5, 1.0] (4)
±Δ*a*	0.02	0.02	0.02	0.02	0.02	±[0.0, 0.1] (4)

Only two seed parameters (shown in red) were varied among species to facilitate the interpretation of results.

(1) field experiment; M. Sternberg, unpubl. data.

(2) Allen PS, Meyer SE, Khan MA: **Hydrothermal time as a tool in comparative germination studies.** In *Seed Biology: Advances and Applications* Edited by Black M, Bradford KJ, Vázquez-Ramos J. Wallingford, U.K.: CAB International; 2000:401-410. σ is the aggregated standard deviation of psammophytes and generalist species.

(3) Köchy M, Tielbörger K: **Hydrothermal time model of germination: parameters for 36 Mediterranean annual species based on a simplified approach.** *Basic and Applied Ecology* 2007, **8**:171-182 (doi:10.1016/j.baae.2006.04.002).

(4) derived from Goldberg DE, Turkington R, Olsvig-Whittaker L, Dyer AR: **Density dependence in an annual plant community: variation among live history stages.** *Ecological Monographs* 2001, **71**:423-446.
