# Supplementary material for: Effects of simulated daily precipitation patterns on annual plant populations depend on life stage and climatic region
Source: BMC Ecol. 2008 Mar 27;8:4. doi: 10.1186/1472-6785-8-4 (PMC2359731; doi:10.1186/1472-6785-8-4)
Supplement: Additional file 3 — Standard parameters used in the plant module. Standard parameters used in the plant module for all simulations. [file 1472-6785-8-4-S3.pdf]

Standard parameters used in the plant module.

climate	arid	semi-arid	dry Mediterranean	typical Mediterranean	mesic Mediterranean	range in sensitivity analysis
representative species (for illustration only)	*Stipa capensis*	*Crithopsis delileana*	*Trisetaria macrochaeta*	*Avena sterilis*	*Brachypodium dystachium*	
PWP (MPa)	–6.5 (1)	–5.5 (1)	–3.5 (1)	–1.75 (1)	–1.5 (1)	[–7.5, –1.5]
m_{seed} (mg)	3	3	3	3	3	[0.1, 5.0] (2)
m_{fruit} (mg)	3.81	3.81	3.81	3.81	3.81	[1.0, 2.0]· m_{seed} (7)
RGR_{max}	0.17	0.17	0.17	0.17	0.17	[0.1, 0.2] (2)
competitiveness C (%)	20	20	20	20	20	[10, 50]
$m_{established}$ (mg)	5.0	5.0	5.0	5.0	5.0	[1.5, 2.5]· m_{seed}
m_{A2} (mg)	3.5	3.5	3.5	3.5	3.5	[1.1, 1.5]· m_{seed}
m_{A3} (mg)	4.5	4.5	4.5	4.5	4.5	[1.1, 1.5]· m_{A2}
m_{A4} (mg)	6.5	6.5	6.5	6.5	6.5	[1.1, 1.5]· m_{A3}
m_{max} (mg)	500	500	500	500	500	[100, 1500]
m_{mature} (mg)	20	35	35	40	40	[0.05, 0.2]· m_{max}
m_{C1} (mg)	30	30	30	30	30	[15, 50]
m_{C2} (mg)	55	55	55	55	55	[1.5, 4]· m_{C1}
allocation (%)	60	60	60	60	60	[30, 80] (4, 5, 6)
density-dependent mortality, M_D (%)	40	40	40	40	40	[20, 60] (3, 8)

Only two plant parameters (shown in red) were varied among species to facilitate the interpretation of results.

(1) derived from relative species abundance at the field sites (I. Konsens, pers. comm.) and Majerus ME: **Response of root and shoot growth of three grass species to decreases of soil water potential.** *Journal of Range Management* 1975, **28**:473-476.

(2) Dyer AR, Turkington R, Goldberg DE, Sayre C: **Effects of growing conditions and source habitat on plant traits and functional group defintion.** *Functional Ecology* 2001, **15**:85-95.

(3) derived from Goldberg DE, Turkington R, Olsvig-Whittaker L, Dyer AR: **Density dependence in an annual plant community: variation among live history stages.** *Ecological Monographs* 2001, **71**:423-446.

(4) Aronson JA, Kigel J, Shmida A: **Reproductive allocation strategies in desert and Mediterranean populations of annual plants grown with and without water stress.** *Oecologia* 1993, **93**:336-342.

(5) Grünzweig JM, Körner C: **Biodiversity effects of elevated CO_2 in species-rich model communities from the semi-arid Negev of Israel.** *Oikos* 2001, **95**:112-124.

(6) Gonzalez Ponce R, Santin I: **Competitive ability of wheat cultivars with wild oats depending on nitrogen fertilization.** *Agronomie* 2001, **21**:119-125.

(7) Kigel J, Perevolotsky A, Roundy BA, Allen P, Mayer SE: **Predicting seed-bank germination in semiarid rangelands under grazing. Final report. International Arid Lands Consortium Project 98R-27, Research Grant 191-1-610-8. Final Report.** 2002.

(8) unpublished field data (H. Parag, pers. comm.)
