# Supplementary material for: Effects of simulated daily precipitation patterns on annual plant populations depend on life stage and climatic region
Source: BMC Ecol. 2008 Mar 27;8:4. doi: 10.1186/1472-6785-8-4 (PMC2359731; doi:10.1186/1472-6785-8-4)
Supplement: Additional file 4 — Absolute changes of variables. Alternative view of data shown in Fig. 2 based on relative changes of mean annual precipitation. [file 1472-6785-8-4-S4.pdf]

Additional file 4 — 'Relative' changes of variables

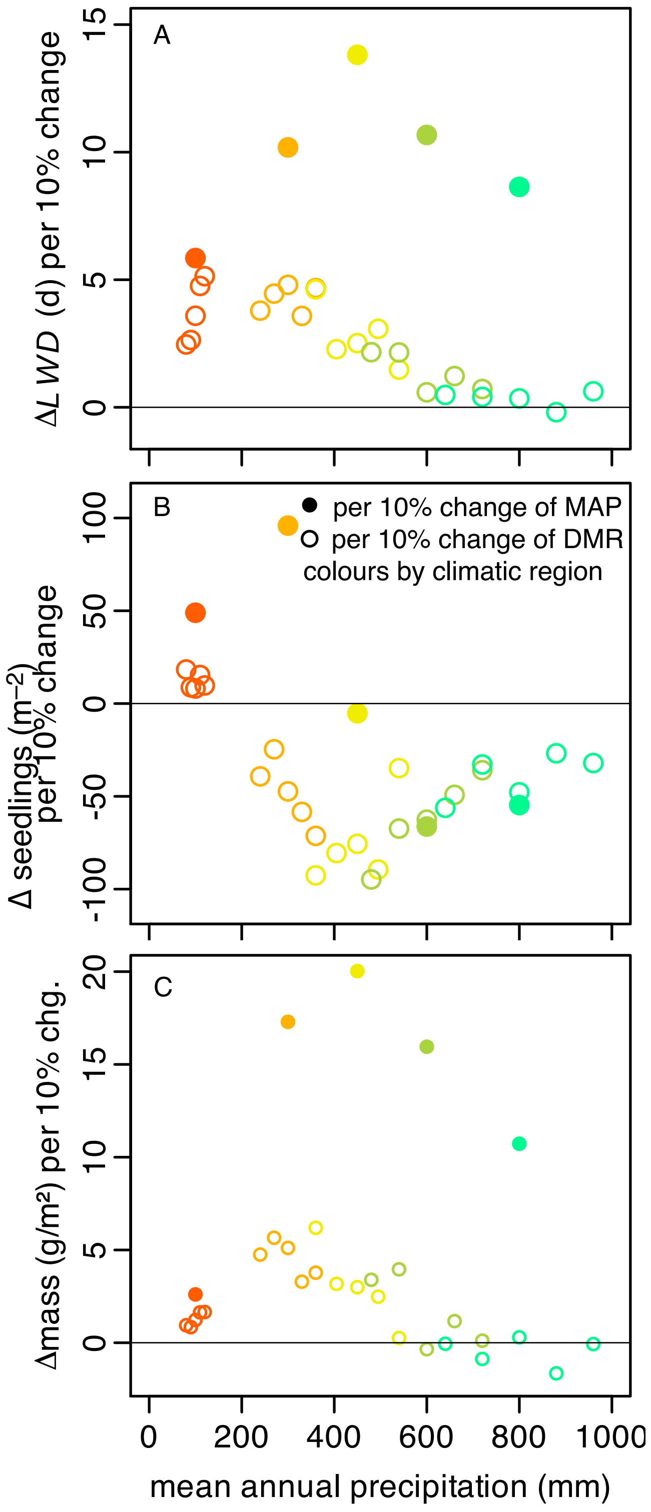

Fig. A4.1. Changes of variables expressed per 10% change of MAP (slope of black lines of Fig. 2 A, C, E) [dots, ●] and, for comparison, changes of variables per 10% change of DMR (slope of thin coloured lines of Fig. 2 B, D, F) [circles, ○]. This shows the effect of the relative change of DMR and MAP as expected for the cited climate change scenarios. Colours correspond to different climatic regions (red: arid, orange: semiarid, yellow: dry Mediterranean, green: typical Mediterranean, bluegreen: mesic Mediterranean).
