# Supplementary material for: Effects of simulated daily precipitation patterns on annual plant populations depend on life stage and climatic region
Source: BMC Ecol. 2008 Mar 27;8:4. doi: 10.1186/1472-6785-8-4 (PMC2359731; doi:10.1186/1472-6785-8-4)
Supplement: Additional file 5 — Precipitation distribution at climate stations. Precipitation distribution at climate stations close to the field sites. [file 1472-6785-8-4-S5.pdf]

Overview of climatic variables of stations near the field sites.

climate station	longitude (° E)	latitude (°N)	years	annual precipitation (mm)		days per year with precip. >5 mm			days per year with precip. >15 mm			days per year with precip. >25 mm		
				mean	CV	mean	SD	interval	mean	SD	interval	mean	SD	interval
Sede Boqer	34.78	31.23	1952-2000	89	47	6	3	18	1	1	25	0	1	39
Be'er Sheba	34.90	31.62	1957-2000	196	36	12	4	7	3	2	32	1	1	20
Beth Guvrin	35.03	31.80	1950-2000	395	34	22	6	3	9	4	12	4	2	19
Rosh Zurim	35.22	33.05	1983-2000	567	38	26	7	3	13	5	7	7	4	14
Beth Meir	35.50	32.97	1977-2000	625	32	29	7	3	14	5	7	8	4	17
Har Kena'an	34.79	30.87	1940-1999	717	26	36	8	2	16	5	7	8	3	12
Elon	35.45	33.07	1974-2000	813	26	38	8	2	19	5	6	9	4	14

SD: standard deviation, CV: coefficient of variation. 'interval' is the mean across years of the median interval between days with the respective amount of rain.
