# Supplementary material for: Effects of simulated daily precipitation patterns on annual plant populations depend on life stage and climatic region
Source: BMC Ecol. 2008 Mar 27;8:4. doi: 10.1186/1472-6785-8-4 (PMC2359731; doi:10.1186/1472-6785-8-4)
Supplement: Additional file 6 — Model source code. The archive contains directories with the source code in the c++ language distributed over several text files. The archive also includes a sample input file for running the model in batch mode. [file 1472-6785-8-4-S6.zip › AnnualsModelSourceCode/DOCUMENTATION.rtf]

The computer code is written in C++ and compiles with the gcc 3.3 compiler.The source code files use either Macintosh or UTF text encoding.Parameters for the simulations can be entered interactively (only main parameters) or by starting the program from the command line with the name of a batch file as start parameter.The directory of files with daily rain data must be entered in main.cpp on line 50/51. Files for batch input must be located in the directory as the program. Batch input files must be tabulator-separated. On Windows computers, the line endings must be Windows-style. If the first line contains column headings, the line must be preceded by a # character (see sample_batch_file.txt). The file must contain the following parameters in this order:#ID	user-defined ID of the parameter combination (integer)trials	number of repeats of one parameter combination (integer)years	number of years to simulate (integer)RFile	name of a file containing daily rain data (char); a sample file "BG.txt" is includedAnnPrecip	modification of mean annual precipitation (float): the daily rain values of each year are divided by the annual sum and multiplied with AnnPrecip. -2 signals that the program should calculate stochastic time series defined by the following six parameters (RH to Vw), -1 signals that the values are not transformedRH	amplitude parameter for rain probability (float)RX	location parameter for rain probability (float)RW	width parameter for rain probability (float)Vh	amplitude parameter for rain volume (float)Vx	location parameter for rain volume (float)Vw	width parameter for rain volume (float)T_mean	mean annual temperature (float)T_var	range of mean monthly temperatures (float)RGRShade	relative reduction of relative growth rate if the plant grows under shrubs (float)TopoV	relative local variation of water infiltrating the soil if the infiltrating water < 10 mm (float)stones	relative cover of surface by stones (float)stones_var	variation of "stones" (float)shrub_cover	total shrub cover (%) across the lattice (float)shrub_radius	radius of individual shrubs (cm, float)soil parameters, for details see Additional file 1 of the articleevapoO	evaporation factor O layer (float)evapoA1	evaporation factor A layers (float)infRateShrub	infiltration rate under shrubs (float)infRateHerb	infiltration rate under herbs (float)infRateBare	infiltration rate when soil is bare (float)deltaInfRate	change of infiltration rates when soil is saturated (float)permA	permeability of A layers (float)permC	permeanility to C layer (float)depthA4	thickness of A4 layerNSupply	index of N supply (0-3)Runon	should runoff be added to cells? (0,1)alpha	van Genuchten parameter (float)beta	van Genuchten parameter (float)thetaR	van Genuchten parameter (float)thetaS	van Genuchten parameter float)seed parameters, for details see Additional file 2 of the articleconstSB	should the seed bank reset each year? (0,1)seedN	seed bank density (per square metre, float)seedNV	variation of seedN (float)surv	survival fraction of newly produced seeds (float)survV	variation of surv (float)persist	persistence of seed bank (float)DDv	variation of DD (float)DD	factor controlling densitiy-dependent germination (float)PsiB50m	parameter for hydrothermal time model of germination (float)PsiB50b	parameter for hydrothermal time model of germination (float)PsiB50S	parameter for hydrothermal time model of germination (float)thetaH	parameter for hydrothermal time model of germination (float)Tmin	parameter for hydrothermal time model of germination (float)DryDays	parameter for hydrothermal time model of germination (float)maxGerm	maximum germination fraction (float)plant parameters, for details see Additional file 3 of the articleHome	fraction of resources from own cell (float)MA2	mass threshold for access to water from A2 layer (float)MA3	mass threshold for access to water from A3 layer (float)MA4	mass threshold for access to water from A4 layer (float)MC1	mass threshold for competition with primary neighbours (float)MC2	mass threshold for competition with secondary neighbours (float)MSeed	seed mass (float)MFruit	fruit mass (float)MEstablish	mass threshold for plant considered established (float)MFlower	mass threshold for flowering (float)MMax	maximum mass (float)RGRmax	maximum relative growth rate  (float)PWP	permanent wilting point  (float)Allocation	allocation fraction to reproductive tissue (float)NTol	tolerance of low nitrogen supply index (0-3)Mort	mortality  (float)Parameters that are meaningful to change reside in the directory "Parameter".The program includes functions for growth of annuals under shrub cover but these have not yet been validated.The directory for files containing daily rain values must be specified on line 50 of file main.cpp. The format of these files is described in file rain.h.
