# Supplementary material for: Effects of simulated daily precipitation patterns on annual plant populations depend on life stage and climatic region
Source: BMC Ecol. 2008 Mar 27;8:4. doi: 10.1186/1472-6785-8-4 (PMC2359731; doi:10.1186/1472-6785-8-4)
Supplement: Additional file 7 — Validation. The file describes how the soil, seed bank, and plant modules of the model were validated. The file contains text and three figures. [file 1472-6785-8-4-S7.pdf]

Validation

Model parameters (see additional file 1: ParametersSoil.pdf, additional file 2: ParametersSeedBank.pdf, additional file 3: ParametersPlants.pdf) were derived from field data in the 2002/2003 season, published sources, and expert knowledge. Parameters for which no estimates were available were tuned to fit the temporal course of soil moisture or the seedling density, density of adult individuals, and peak shoot mass in each field site. The simulated soil moisture dynamics, seedling density, and vegetation production was validated by comparison with field measurements in the 2003/2004 season that were not used to parameterize the model. In addition, I compared the simulated with the observed relationships between seedling density and annual rain and between shoot mass and rain.

Soil water potential

The simulations for validation used daily rainfall of the 2003/2004 season of each site as input. I compared the model output of soil water potential in the A1 layer (Ψ_{A1}) to the corresponding values measured in the field in the 2003/2004 season (Fig. A7.1). Ψ_{A1} was calculated from volumetric soil moisture measured by two calibrated TDR sensors. The daily data of these sensors were significantly and strongly correlated at each site, ranging between $r = 0.87$ and 0.99. The correlation of modelled soil moisture with either of the two sensors was also significant and strong, ranging between $r = 0.77$ and 0.90.

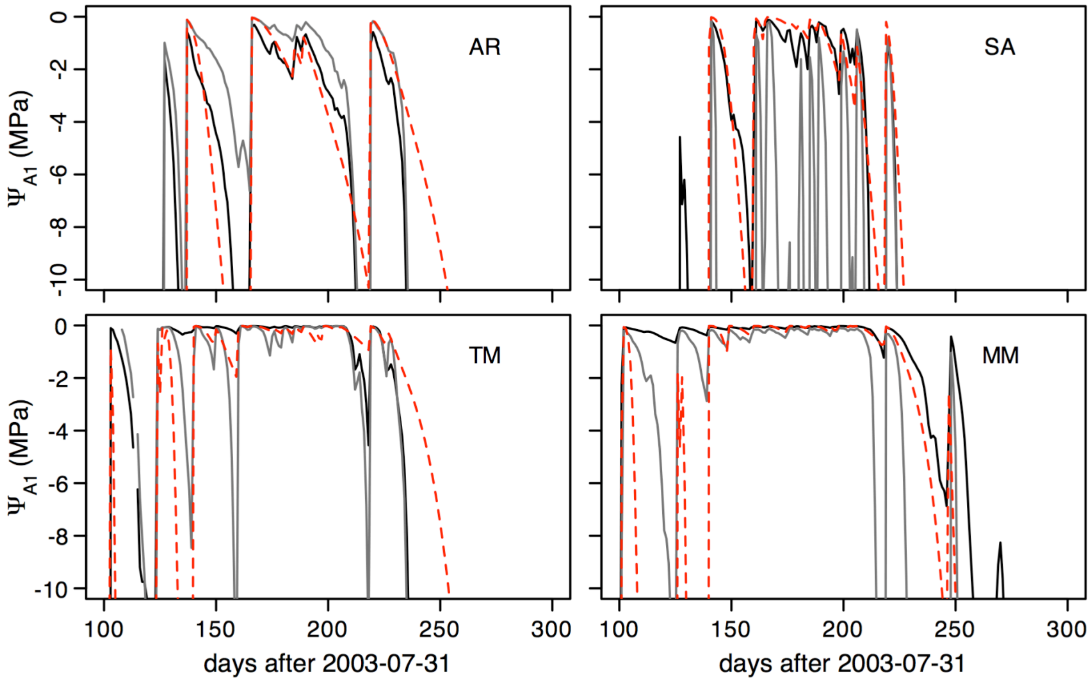

Fig. A7.1 – Comparison of simulated with measured soil water potential.

Panels: AR: arid, SA: semiarid, TM: typical Mediterranean, MM: mesic Mediterranean site. Red, dashed lines: simulated soil water potential in the A1 layer (1–5 cm) using 2003/2004 rain data as model input. Black and grey continuous lines: soil water potential calculated from measurements with two TDR sensors in 2003/2004.

Seedling density and peak shoot mass per site

I compared the model output for seedling density and peak shoot mass with the corresponding values measured in plots in the field in the 2003/2004 season (Fig. A7.2). Seedling density was averaged across 5 replicates in each of five control plots. For mass only averages for all control plots were available. The model used daily rainfall and average seed bank density of the 2003/2004 season of each site as input. Simulations were conducted for 5 plots × 5 years and values averaged across years. Simulated seedling density for 2003 was within the range of values observed in the field but had a much smaller variability (Fig. A7.2a). Apparently, variability of the seed bank in 2002/2003, which was used for parameterization of the model was smaller than in 2003/2004. The simulated averages of peak shoot mass were close to those observed at the field sites (Fig. A7.2b, A7.3b). The sensitivity analysis (additional file 8: SensitivityAnalysis.pdf) suggested that average growth rate or seed mass may have been higher in 2003/2004 than in the preceding year causing a slight underestimation of actual values by the model.

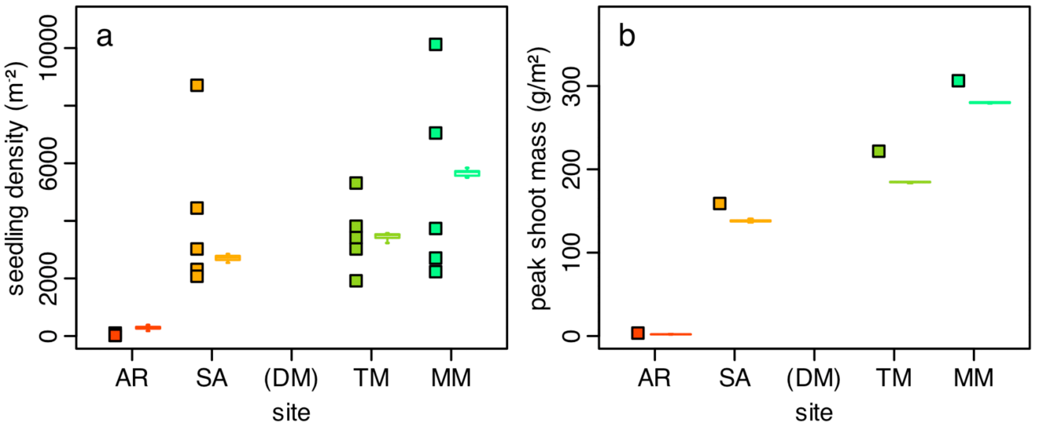

Fig. A7.2 – Comparison of simulated with measured values in 2003/2004. Measured (squares) and simulated values (box plots) of a) seedling density and b) peak shoot mass in 2003/2004. The simulation used the 2003/2004 rain data and seed bank density and was parameterized with values from 2002/2003. Box plot whiskers indicate the distance of 1.5 · the interquartile range (box height). Colours correspond with sites.

Relationship of seedling density and peak shoot mass with precipitation amount

In addition to the comparison of values for the 2003/2004 season at individual sites with specific environmental conditions, I compared the simulated with the observed patterns of seedling density and peak shoot mass as a function of precipitation amount. For the comparison I used simulated values of 5 years × 10 replications and averages per plot from the control, drought, and irrigation treatments at the field sites from 2002 to 2004 (Fig. A7.3). For mass, I included available data from the 2001/2002 season, but for all years I had access only to treatment averages. The simulations applied the dynamic seed bank scenario and used historic rainfall data

with varying annual volumes (except the semi-arid site for which I could not obtain historic data; instead I used a stochastic time series for 300 mm [20]).
Simulated seedling density increased with annual rain, whereas no obvious trend existed across plots and treatments in the field (Fig. A7.3a). Nonetheless, simulated seed bank density was within the range of observed values. Since the model can simulate seedling density well when seed bank density is known, the lack of correspondence can be attributed to the fact that the simulations used seed bank densities averaged across treatments and years.
Simulated peak shoot biomass corresponded well with observed values. The 95%-confidence bands of the regressions on log-transformed rainfall values overlapped, indicating that the model is capable of reproducing the observed pattern across the whole rainfall gradient.

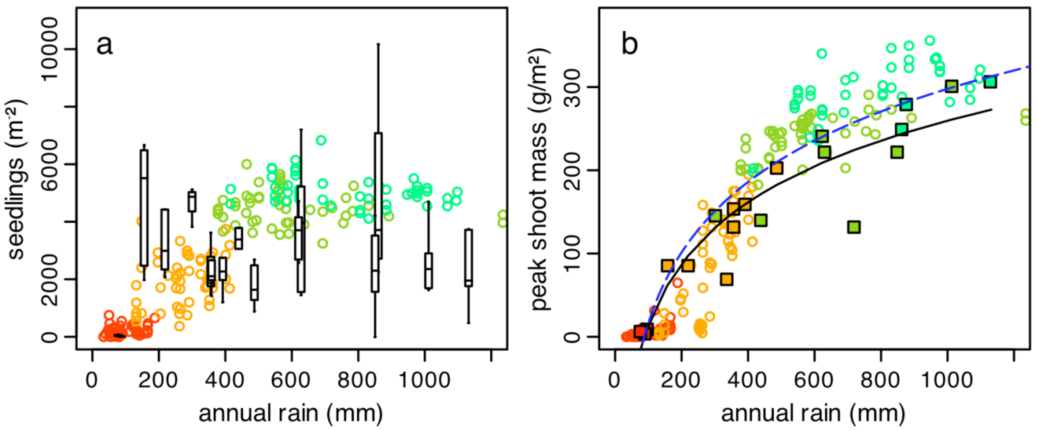

Fig. A7.3 – Comparison of simulated with observed relationships with annual precipitation.

Observed (boxplots, squares, solid line) and simulated (circles, dashed line) patterns of a) seedling density and b) peak shoot mass in relation to annual precipitation. Box plot whiskers indicate the distance of 1.5 · the interquartile range (box height). Confidence bands of the regressions of mass on rain amount overlapped. Colours correspond with sites.
