# Supplementary material for: Effects of simulated daily precipitation patterns on annual plant populations depend on life stage and climatic region
Source: BMC Ecol. 2008 Mar 27;8:4. doi: 10.1186/1472-6785-8-4 (PMC2359731; doi:10.1186/1472-6785-8-4)
Supplement: Additional file 8 — Sensitivity analyses. The file describes the method and results of testing the sensitivity of runoff, soil moisture, and peak shoot mass to changes in parameters. The file contains text and two figures. [file 1472-6785-8-4-S8.pdf]

Sensitivity analyses

The variation of mechanisms in the model allows making inferences about the importance of the mechanisms (structural sensitivity). To aid the interpretation of my results I conducted simulations with a linear instead of the roughly reciprocal soil moisture retention curve, excluded competition among seeds, or excluded competition among established plants. Results of these simulations are mentioned in the relevant paragraphs of the *Discussion*.

To investigate how strongly individual parameters affect the results of the simulations I varied parameters within a plausible range. If the outcome of a simulation is highly sensitive to a change in the value of a parameter, the value must be determined carefully. High sensitivity also suggests that the parameter is involved in important mechanisms controlling the value of dependent variables. I carried out two separate sensitivity analyses: one for climate and soil parameters and one for seed bank and plant parameters. I used Latin hypercube sampling [1, 2] to choose 1000 uniformly distributed subsamples of the parameter space. Each subsample was simulated for 5 time steps in a constant seed bank scenario. The range of the parameters was chosen so that they included a reasonable range of possible conditions across the regions. The standardized partial regression coefficient (*B*) was used to assess the sensitivity to dependent variables selected by stepwise regression. Before the regression, I used the best Box-Cox transformation of the dependent variables, averaged across years within a subsample, to homogenize the variances for the regression.

Climate and soil parameters

The dependent variables analyzed for sensitivity were runoff (mm), mean annual soil moisture (v%) in the A1 layer, representing the moisture resource for seedlings, mean annual soil water potential in the A3 layer (Ψ_{A3}) representing the moisture conditions for most mature plants, and, as an integrative and important factor for plant growth, the longest wet period (*LWP*, the maximum number of consecutive moist days with $\Psi > -3$ MPa). The parameter space for the climate module was defined by the following intervals: mean annual precipitation $R = [50, 200]\% \cdot$ mean annual precipitation of each region, mean annual temperature $T = [15, 20]$ °C, and range of mean monthly temperature $\Delta T = \pm[5, 10]$ K. Those for the soil module are specified in additional file 1: ParametersSoil.pdf. The analysis was calculated with the daily rainfall data of each corresponding field site from August 2002 to July 2003. Plant growth was turned off for sensitivity analysis of the soil module, because I was interested in the direct response of the soil without transpiration effects of the vegetation.

The ranking of absolute standard partial regression coefficients with $|B| > 0.1$ was mostly consistent across regions for runoff, soil moisture in the top A layer, and water potential in the A3 layer (Fig. A8.1), although the absolute *B* values increased or decreased with the regions' aridity. Runoff was most sensitive to rain volume (mean $B = 0.73$) and infiltration rate (mean $B = -0.45$). Soil moisture in the A1 layer was most sensitive to van-Genuchten parameter β (mean $B = -0.64$) describing the moister part of the soil moisture retention curve and the residual water content θ_R (mean $B = 0.60$). Water potential in the A3 layer (Ψ_{A3}) was also most sensitive to β (mean $B = -0.67$), but the second most sensitive parameter was A-layer hydraulic conductivity at all regions except the arid one, where rain volume was the second most sensitive parameter. The sensitivity of the longest wet period LWP varied strongly among

regions (Fig. A8.1). In the arid region, *LWP* was most sensitive to β (B = 0.51) and rain volume (B = 0.30), whereas conductivity and depth of the A layer had the strongest effects on *LWP* in the other regions. The effect of β was positive in the arid and semi-arid regions, but negative in the two Mediterranean regions. Evaporation from the surface and temperature never had a strong effect on either of the dependent variables. The tested parameters accounted for >80% of the variability of runoff, soil moisture in the top A layer, and water potential in the A3 layer, but only 15–47% of the variation of *LWP*. In summary, simulated soil moisture was most sensitive to the two soil-texture related parameters β and conductivity, and rain volume. Infiltration rate had a strong effect on runoff, which would have a feedback on the inflow of water to the soil. Therefore, simulation of plant growth can also be expected to vary with soil type.

Seed bank and plant parameters

In this analysis I investigated the sensitivity of peak shoot mass to seed bank, germination, and growth parameters (additional file 2: ParametersSeedBank.pdf, additional file 3: ParametersPlants.pdf). The variability of annual rainfall (provided as a stochastic time series) was included in the analysis for reference but not included in the Latin hypercube of parameters. Since peak shoot mass was more variable than soil moisture, I executed four replicate model simulations for each parameter combination instead of a single replicate. Soil and climatic conditions were derived from measurements at the four field sites.

Between 71 and 82% of the variation in mass was explained by the tested parameters. The ranking of absolute standard partial regression coefficients varied with aridity of the region (Fig. A8.2). Thus, water-related parameters (annual rain volume, PWP, and germination parameters θ_{HT}, Ψ_{b50}, and $\sigma(\Psi b)$) had stronger effects in the two arid than in the two Mediterranean regions. In contrast, parameters related to plant performance (density-dependent germination *a*, mass for a plant to compete with immediate neighbours m_{C1}, maximum shoot mass m_{max}, competitiveness C, and RGR) had greater effects in the Mediterranean regions than in the more arid regions. Seed bank size had a consistent, intermediate effect across all regions.

The sigmoid form of the shoot mass–rain relation may be suspected to be caused by the specification of a logistic growth rate, which also describes a sigmoid relationship. Test simulations with a constant growth rate (exponential growth), however, showed that the shoot mass curve remains sigmoid. Rather, the sigmoid shape was linked to *LWP* that was also a sigmoid function of annual rain volume.

References

1. Helton JC, Davis FJ: **Latin hypercube sampling and the propagation of uncertainty in analyses of complex systems.** *Reliability Engineering and System Safety* 2003, **81**:23-69.

2. Minasny B: **Latin hypercube sampling** [http://www.mathworks.com/matlabcentral/fileexchange/loadFile.do?objectId=4352&objectType=file]; viewed 2004-11-01.

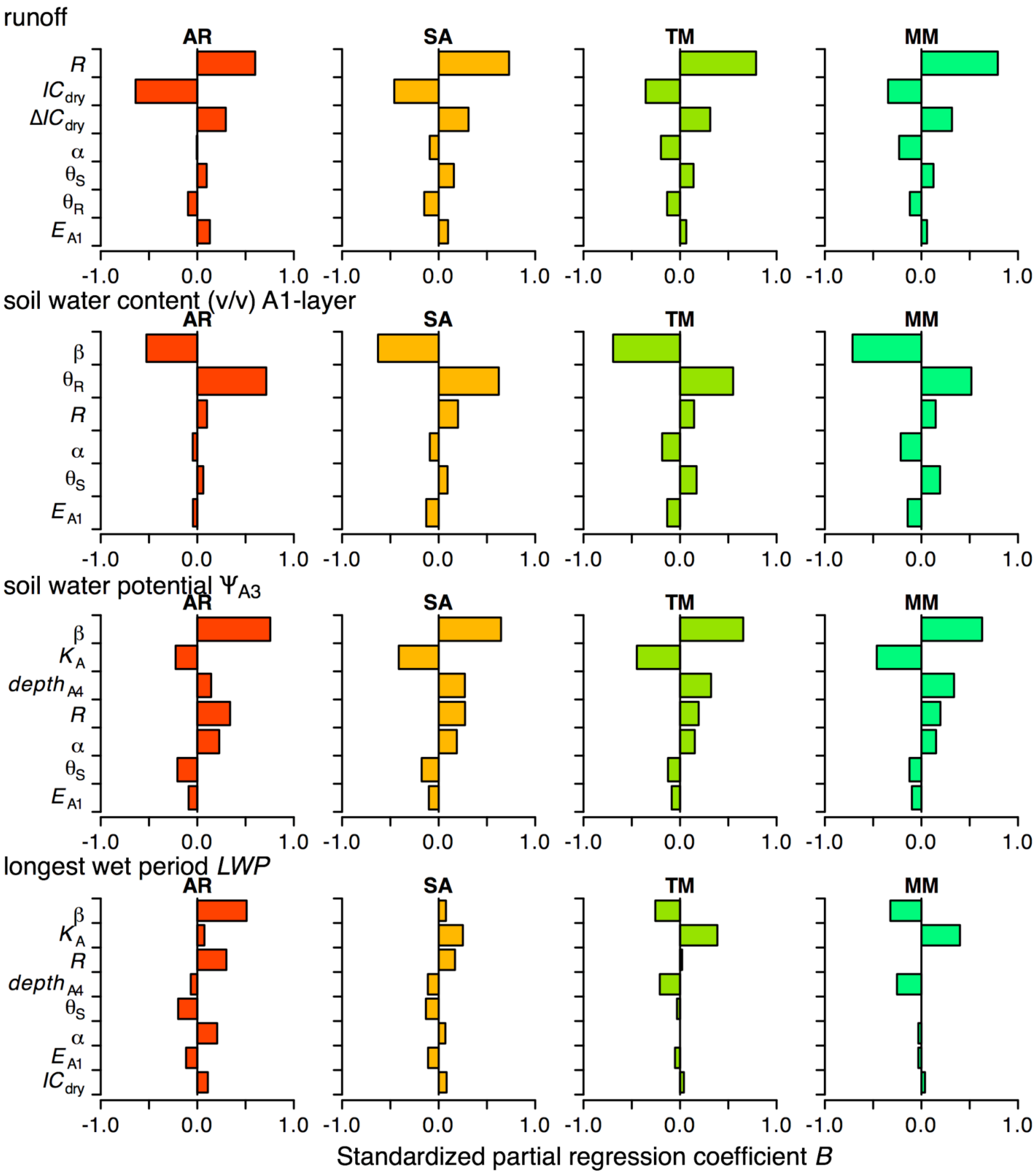

Fig. A8.1 – Sensitivity of simulated soil variables to variation of model parameters.

Sensitivity of runoff, soil water content in the A1 layer, soil water potential at 10–15 cm depth (Ψ_{A3}), and the longest wet period (*LWP*, annual maximum number of continuous days that at least one A layer has $\Psi > -3$ MPa) to 15 parameters. Sensitivity was measured by the standardized partial regression coefficient (*B*). Only parameters with $|B| > 0.1$ are presented. $depth_{A4}$: depth of the A layer, E_{A1}: evaporation factor for A1 layer, IC_{dry}: infiltration coefficient for dry soil, K_A: hydraulic conductivity of A layer, *R*: percent change of mean annual precipitation, ΔIC_{dry}: variation of IC_{dry}, α, β, θ_R, θ_S: parameters of the van-Genuchten soil moisture retention curve. The parameters are ordered by their average rank across the climatic regions.

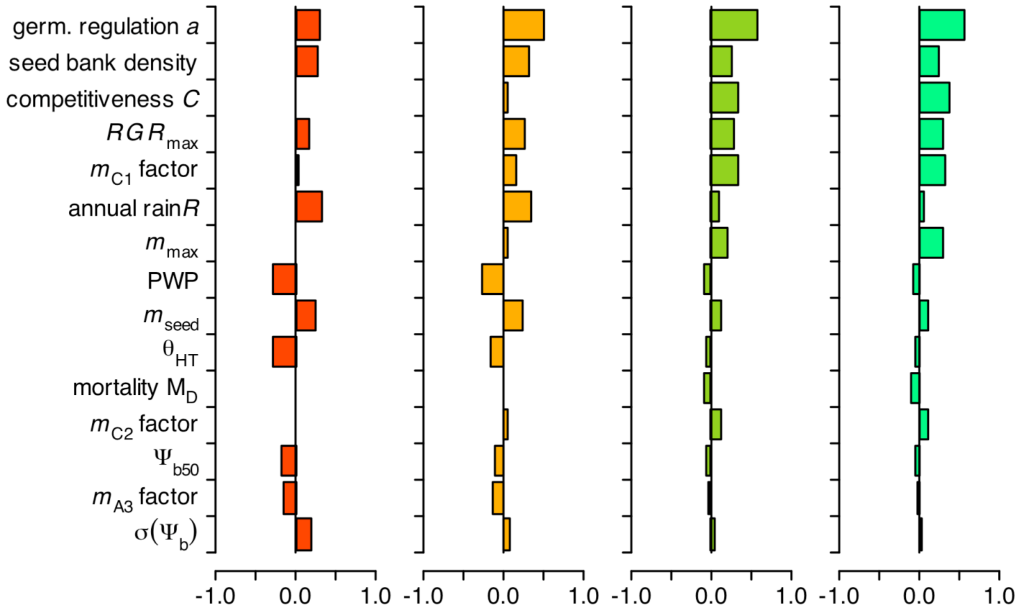

Fig. A8.2 – Sensitivity of simulated plant variables to variation of model parameters.

Sensitivity of peak shoot mass (g/m^2) to 22 parameters (standard partial regression coefficient, B) for four regional scenarios. Only parameters with $|B| > 0.1$ for at least one region are shown. a: density regulation of germination, m_{C1}: factor multiplied with m_{seed}, the product is the minimum mass for competition with immediate neighbours, m_{C2}: factor multiplied with m_{seed}, the product is the minimum mass for competition with secondary neighbours, m_{mature} factor: factor multiplied with m_{seed}, the product is the minimum mass for allocation to reproductive mass, m_{max}: maximum shoot mass, m_{seed}: seed mass, PWP: permanent wilting point, RGR_{max}: maximum relative growth rate, θ_{HT}: hydrothermal time threshold for germination, $\sigma(\Psi_b)$: variation of minimum soil water potential for germination, Ψ_{b50}: median of minimum soil water potential for germination. The parameters are ordered by their average rank across the climatic regions.
